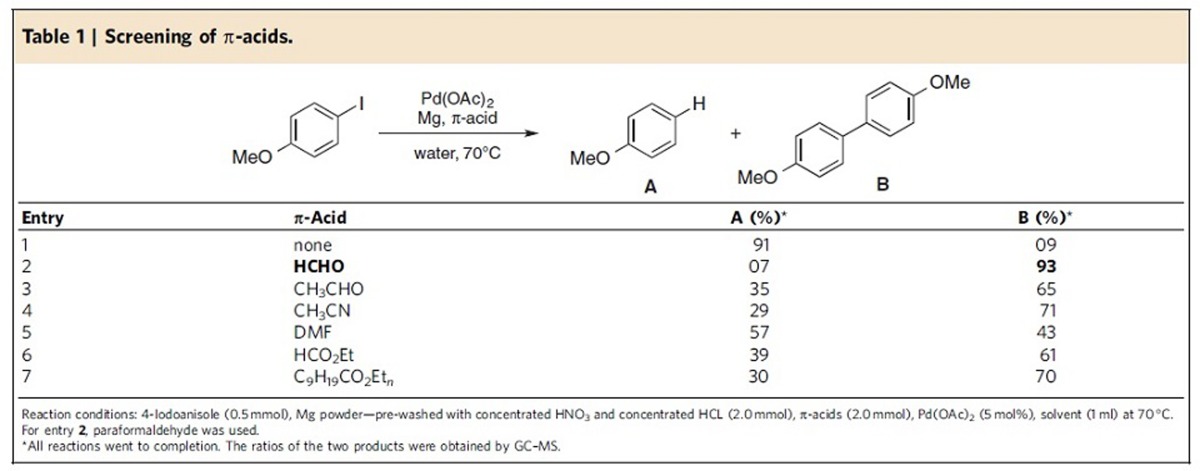# Kumada–Grignard-type biaryl couplings on water

**DOI:** 10.1038/ncomms8401

**Published:** 2015-06-18

**Authors:** Anish Bhattacharjya, Piyatida Klumphu, Bruce H. Lipshutz

**Affiliations:** 1 Department of Chemistry and Biochemistry, University of California, Santa Barbara, California 93106, USA

## Abstract

Well-established, traditional Kumada cross-couplings involve preformed Grignard reagents in dry ethereal solvent that typically react, e.g., with aryl halides via Pd catalysis to afford products of net substitution. Therefore, in the work described, which appears to be counterintuitive, exposure of these same aromatic halides to catalytic amounts of Pd(II) and excess magnesium metal in pure water leads to symmetrical/unsymmetrical biaryls, indicative of a net Kumada-like biaryl coupling. Evidence is presented suggesting that Grignard reagents, formed *in situ* in water, may be involved.

## Abstract (web summary)

Organomagnesium reagents have been a staple for carbon–carbon bond formations for over a century, but are notoriously sensitive to water. Here, the authors show a Kumada-type coupling of aryl halides on water, with a potential intermediary of an *in situ* formed Grignard reagent.

## Graphical abstract

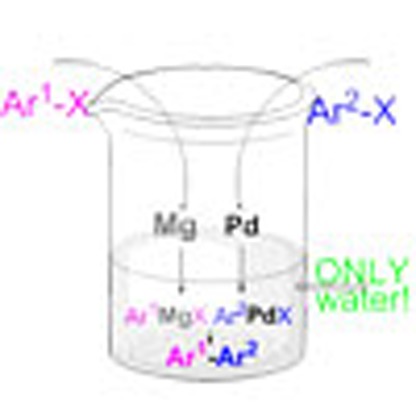

Textbook teachings on the use of highly reactive organometallic reagents, such as organozinc reagents used in Negishi couplings, or Grignard reagents associated with Kumada couplings, are unequivocal: moisture must be avoided^1^. Notwithstanding such common dogma, we have shown that *in situ* generation of organozinc halides from precursor halides, localized within nanomicelles, can lead to water-sensitive species RZnX formed presumably on the surface of added zinc metal^2^,^3^,^4^,^5^,^6^. The lifetime of RZnX is sufficient to allow for transmetalation to a Pd(II) intermediate, ultimately leading to net Negishi-like couplings in water. While advances have been made using related formation of zinc reagents from precursor aryl halides in water, such as the recent report on Barbier-type 1,2-additions of aryl iodides to aldehydes^7^, the corresponding Kumada-like couplings in water remain a most challenging goal.

We herein disclose that biaryl homo- and hetero-couplings can now be realized using aryl halides in the presence of catalytic palladium and excess magnesium metal with water as the only reaction medium (Fig. 1) (for alternative routes to biaryls going through Ni catalysis and Mn metal, see ref. 8 and for alternative routes to biaryls going through Ni catalysis and Zn metal, see ref. 9). Initial mechanistic studies indicate the involvement of a carbanion-like intermediate.

## Results

### Optimization

The combination of 5–10 mol% Pd(OAc)_2_, along with an excess of either fresh magnesium, or of which the surface has been carefully cleaned, leads in water to rapid reduction of Pd(II) to Pd(0) as confirmed by X-ray photoemission spectroscopy (Supplementary Figs 30,31) forming what appears to be palladium nanoparticles. Heating this mixture of the resulting active catalyst and remaining Mg metal together with either a single aryl halide, or two aryl halides, to 70 °C results in either the homo- or hetero-cross-coupling product(s), respectively. Initially, low yields of the biaryl were obtained, with most of the material being converted to the corresponding protio-quenched arene. However, in the presence of either paraformaldehyde (for homocouplings), or commercially available aqueous formalin (for heterocouplings) as the medium, yields of the biaryl improved substantially. Presumably, this additive serves to assist reductive elimination from a Pd(II) intermediate via ligation as a π-acid^10^. A brief survey of alternative π-acids (Table 1) revealed formaldehyde to be the most effective in producing the desired biaryl (entry 2). The presence of a designer surfactant (for example, TPGS-750-M^11^ or Nok^12^) had no effect.

### Scope of homocoupling

**Figure 2:**
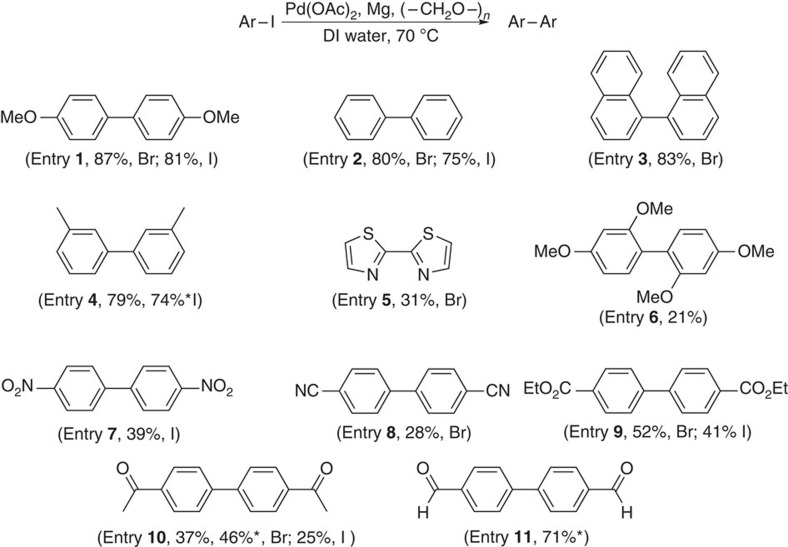
Homocouplings of aryl halides in water. Reaction conditions: aryl halide (0.5 mmol), Mg powder—pre-washed with concentrated HNO_3_ and concentrated HCl (2.0 mmol), paraformaldehyde (2.0 mmol), Pd(OAc)_2_ (5 mol%), DI, deionized water (1 ml) at 70 °C. The yields reported are for products isolated and purified by flash chromatography. *The reactions were done with (unwashed) Mg obtained from a freshly purchased bottle.

Several aryl bromides and iodides were subjected to these aqueous homocoupling conditions, as illustrated in Fig. 2. The following trends were noted: (1) aryl bromides uniformly afforded higher isolated yields of biaryl products (compare Fig. 2, entries **1**, **2**, **9** and **10**); (2) aryl halides bearing electron-withdrawing groups (Fig. 2, entries **7**–**10**) afforded modest to poor results, although the case of *p*-bromobenzaldehye was the exception (Fig. 2, entry **11**); (3) electrophilic residues on the aromatic ring (Fig. 2, entries **8**–**11**) did not lead to products resulting from 1,2-addition; (4) lesser amounts of formaldehyde, or reduced temperatures (for example, room temperature or 50 °C) led to lower yields and increased levels of protio-quenched arenes; (5) use of stoichiometric amounts of reduced Pd(OAc)_2_, in the absence of Mg, led to no homocoupling product.

### Scope of heterocoupling

**Figure 3:**
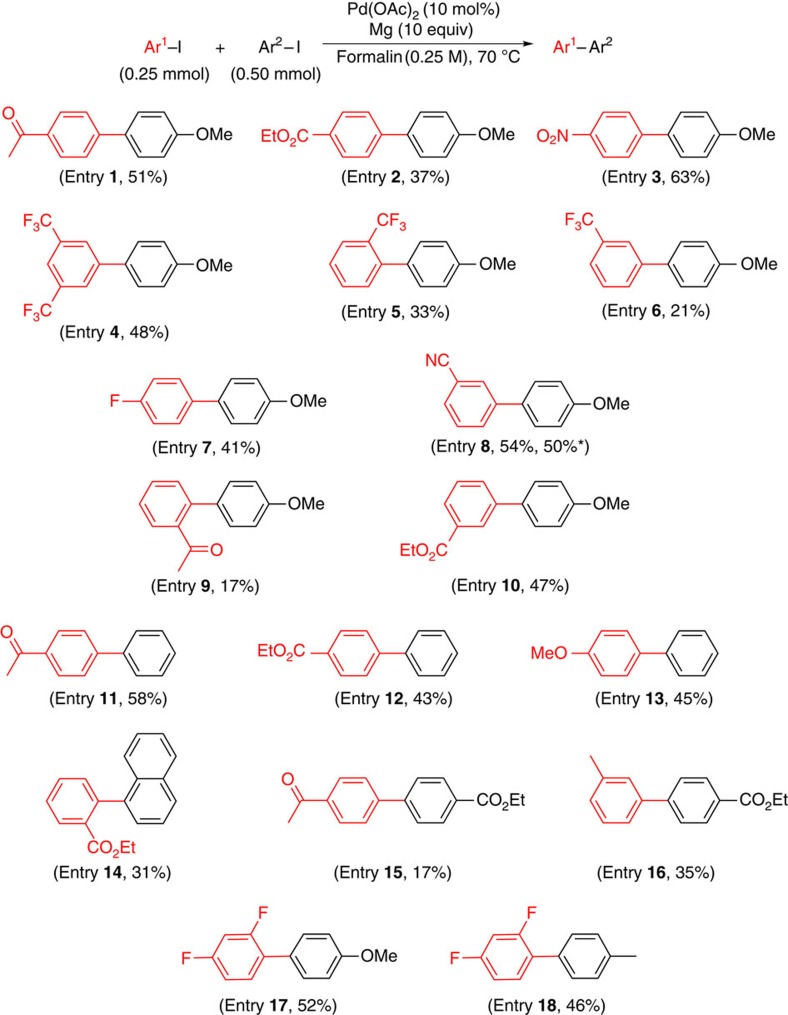
Heterocouplings of aryl halides in water. Reaction conditions: aryl halide (0.25 mmol), Mg powder—pre-washed with concentrated HNO_3_ and concentrated HCl (2.5 mmol), Pd(OAc)_2_ (10 mol%) and formalin (1 ml) at 70 °C. The yields reported are for products isolated and purified by flash chromatography. *The reaction was done with Mg (used directly) from a freshly purchased bottle.

Heterocouplings of aryl iodides were also investigated under these aqueous conditions, with several examples shown in Fig. 3. In general, yields were modest. A higher loading of Pd(OAc)_2_ (10 mol%) was required than that needed for homocouplings, as was additional magnesium. Here again, the combination that appears to be least favourable involves aryl iodides when each partner contains an electron-withdrawing group (for example, Fig. 3, entry **15**). Best results were obtained employing the combination of electron-deficient and electron-rich partners. Formalin is necessary for heterocouplings, while paraformaldehyde is the additive of choice for homocouplings. Insofar as heterocouplings are concerned, the major side reaction associated with electron-deficient systems is protio-quenching, with low levels of homocoupling being seen (0–7%). Thus, the side products of Fig. 3, entry **15**, were acetophenone and ethyl benzoate.

### Mechanistic studies

**Figure 4:**
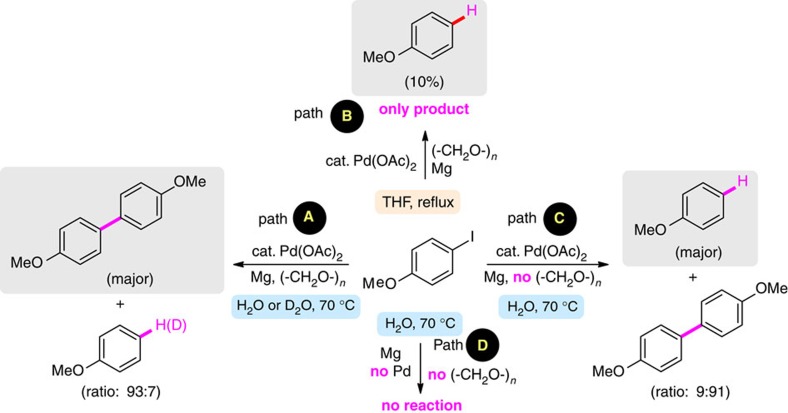
Control reactions. *Reaction conditions: 4-lodoanisole (0.5 mmol), Mg powder—pre-washed with concentrated HNO_3_ and concentrated HCl (2.0 mmol), paraformaldehyde (where applicable, 2.0 mmol), Pd(OAc)_2_ (5 mol%), solvent (1 ml) at 70 °C. ^†^Conversions determined by GC–MS.

Control reactions (Fig. 4) demonstrated that both metals, along with paraformaldehyde, are required for these cross-couplings to occur (path A). Treatment of an aryl halide with magnesium under otherwise identical conditions in tetrahydrofuran (THF) gave a small amount of the protio-quenched arene and no homocoupling (path B). When Pd(OAc)_2_ (5 mol%) plus excess Mg were added, in the absence of formaldehyde, the major product was the same reduced arene (path C). Conducting the coupling in D_2_O gave the anticipated biaryl, while the by-product arene showed 100% deuterium incorporation (path A), confirming water as the source of hydrogen, which is mechanistically significant (*vide infra*).

**Figure 5:**
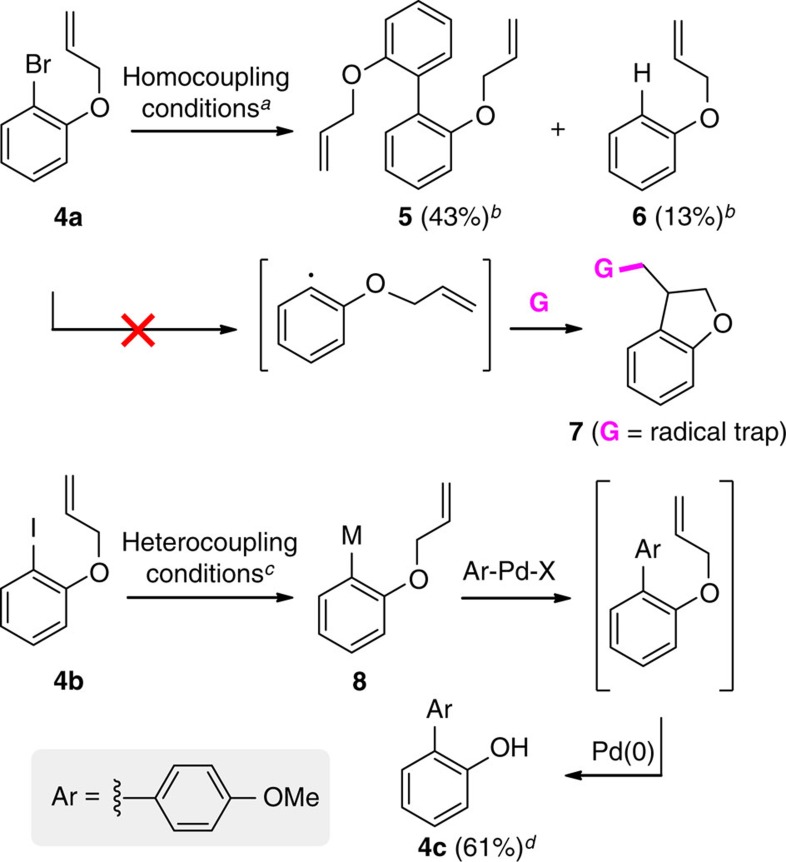
Experiments to test for radical intermediates. *4a (0.5 mmol), Mg powder—pre-washed with concentrated HNO_3_ and concentrated HCl (2.0 mmol), paraformaldehyde (2.0 mmol), Pd(OAc)_2_ (5 mol%) and DI water (1 ml) at 70 °C. **G**=acrylonitrile. ^†^From GC–MS, the rest was phenol coming from de-allylation. ^‡^4b (0.25 mmol), Mg powder—pre-washed with conc. HNO_3_ and concentrated HCl (2.5 mmol), Pd(OAc)_2_ (10 mol%), formalin (1 ml) at 70 °C. ^§^Isolated yield, after purification by flash chromatography.

Additional experiments to assess the potential involvement of free radicals as intermediates in these homo- and hetero-cross-couplings were conducted using *o*-allyloxyhalobenzene^13^ (**4a**; Fig. 5). Under standard conditions, none of the cyclized product **7** was detected by gas chromatography–mass spectrometry. Rather, the products identified were homocoupling product **5** and protio-quenched material **6**. Reaction using the corresponding iodide **4b** and 4-iodoanisole afforded the same two products, although the heterocoupling product was formed in 61% yield, isolated as the deallylated phenol **4c**. The presence of TEMPO interfered with the reaction, as expected given the requirement for electron transfer from Mg metal.

**Figure 6:**
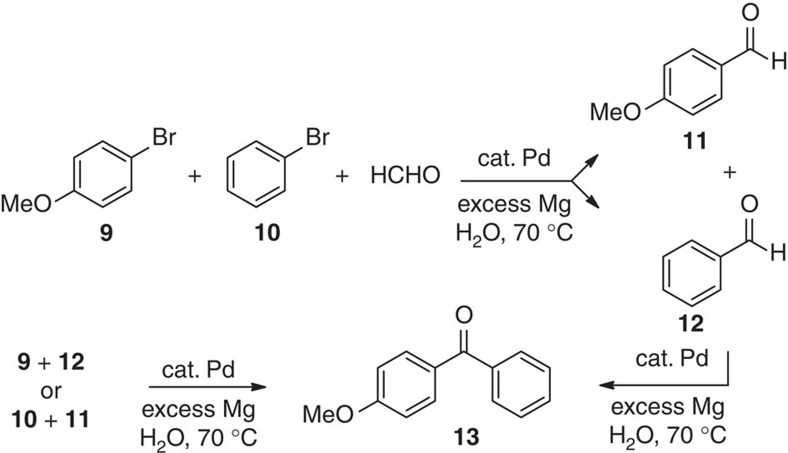
Formation of side products. Aldehydes **11** and **12**, and ketone **13**.

Homo- and hetero-cross-couplings run in the presence of formaldehyde also gave relatively small percentages (0–5%) of aryl aldehydes and diaryl ketones as side products (Fig. 6). These are presumably derived from attack by a carbanionic species on formaldehyde, followed by Oppenauer oxidation of the resulting benzylic alkoxide^14^. The absence of the alcohol can be rationalized by the fact that the alkoxide generated *in situ* is more susceptible to oxidation than the alcohol and hence, is not observed. The diaryl ketone can be similarly formed via subsequent 1,2-addition/oxidation. Control experiments that initiate with an aryl aldehyde (such as **11** or **12**) and aryl halide (such as **9** or **10**), likewise, lead to the same keto product **13**, and support a 1,2-addition/Oppenauer oxidation sequence. Use of greater amounts of formaldehye led to increased levels of aldehyde and ketone side products (Supplementary Table 1).

**Figure 7:**
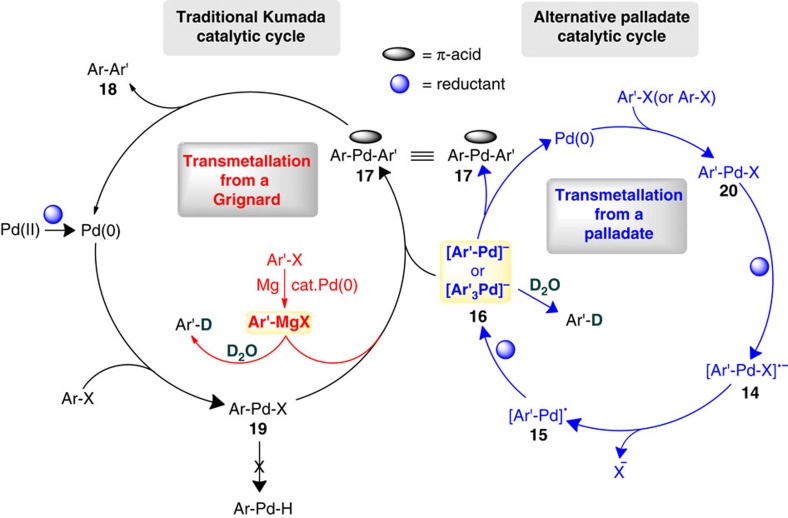
Mechanistic rationale for the generation of biaryl and protio/deuterio-quenched products. Proposed catalytic cycles.

Cross-couplings involving Mg and paraformaldehyde were also examined in which the catalytic Pd(OAc)_2_ was both left out of the mixture, as well as replaced by other metals such as Pt, Ir, Ru and Rh. In the absence of Pd, no reaction was observed. However, in each case with these other metals, the same mixture of products was obtained, albeit in widely varying ratios and with the desired biaryl consistently formed in lower yields. When Mg was replaced with Fe or Zn, the homo- and hetero-coupling products were obtained as well, further suggesting carbanionic intermediates generated from the aryl halide and Mg, Fe or Zn. However, when 20% Pd black (pre-made) was employed in the absence of Mg, no product was obtained indicating that Mg has a more significant role than simply reducing Pd(II) to Pd(0). Such observations indicate that these reactions require (1) a metal in catalytic amounts capable of undergoing oxidative insertion into a C_sp_^2^–X bond, and (2) electrons, coming from Mg, to reduce both the initially present Pd(II), and a Pd–X bond, or for the formation of ArMgX, which then leads to a palladate intermediate (ArPd^−^ and/or Ar_3_Pd^−^, respectively). A mechanistic scheme invoking two potential catalytic cycles to account for the formation of the biaryl product, as well as incorporation of D_2_O, is proposed in Fig. 7.

In the traditional Kumada coupling sequence, both Mg and Pd are required to generate a transient Grignard reagent that is likely to quickly undergo transmetalation to Ar–Pd–Ar′, **17**, or protio/D_2_O quenching. Standard reductive elimination completes the cycle to biaryl Ar–Ar′, **18**. The alternative sequence invokes the obligatory intermediate Ar′–Pd–X (**20**), the reduction of which (likely by Mg°) to **14** then leads via loss of halide ion to radical Ar′–Pd^·^ (**15**). This species is further reduced to anionic palladate **16**, which could exist in either of two anionic forms: Ar′–Pd^−^ or Ar′_3_Pd^−^. Intermediate **19** could then accept an aryl anion from either ArMgX or **16** before reductive elimination via **17**. Species **16** could exist in a (less water-sensitive than ArMgX) resting state for aryl anions, and/or serve as the transmetalating species, transferring an aryl group to Ar–Pd–X (**19**). The lack of coordinating ligands in these Pd-catalysed couplings, however, presents special challenges insofar as a mechanistic picture is concerned^15^,^16^,^17^. The potential role of palladium in the form of nanoparticles^18^, especially under strictly aqueous conditions, adds yet another layer of complexity to this potential alternative catalytic cycle. Clearly, more experimentation is needed to further elucidate these mechanistic issues.

## Discussion

Kumada couplings are among the earliest of reported transition metal-catalysed cross-couplings. They are often preferred over related types of C–C bond constructions when Grignard reagents are readily available, and advantage can be taken of their high reactivity relative to several alternative organometallic partners. However, as with other reactive metal intermediates (for example, RZnX), Grignard reagents are not forgiving in terms of their tolerance of acidic protons and must be used in the complete absence of water, where quenching of these highly basic species is rapid. The option to use a reductive biaryl coupling strategy^19^ involving precursor aryl halides in place of preformed Grignard reagents greatly simplifies the process. Use of water as the reaction medium, contrary to conventional wisdom, further enhances the appeal of this greener process that avoids waste-generating organic solvents. Interestingly, this approach to biaryl formation completely fails upon replacing Pd with Ni (that is, no reaction takes place), indicative of the key electronic match discovered in this study between Pd and Mg in water, which is essential for this chemistry to take place with useful levels of efficiency.

In summary, we have succeeded, for the first time, to achieve Kumada cross-couplings in water. This chemistry involves a fundamental ‘name' reaction traditionally considered extremely unlikely, or even impossible, to accomplish under such conditions. It demonstrates that reactive metal chemistry in water, in this case involving magnesium, can be extended to even more reactive members of the electromotive series, and beyond previously described^2^,^3^,^4^,^5^,^6^ organozinc-mediated couplings. Also of merit is that this new technology potentially opens the door to yet additional synthetic processes that no longer rely on organic solvents, which constitute the majority of organic waste created by the chemistry enterprise^20^,^21^,^22^,^23^. Further studies that extend the scope of these fundamental cross-couplings, run under environmentally responsible conditions and, in particular, that rely on base metals in place of palladium, are underway.

## Methods

### Typical procedure for homocoupling of 4-iodoanisole

To a sample vial (4 ml) equipped with a Teflon-coated magnetic stir bar were added in sequence Pd(OAc)_2_ (25 μmol), magnesium powder (2.0 mmol), 4-iodoanisole (0.5 mmol) and paraformaldehyde (2.0 mmol) (Fig. 2, entry 1). To the mixture was then added deionized water (1 ml). (**Caution!** The water should be added slowly, and the addition should be paused if a vigorous generation of dihydrogen ensues.) The vial was covered with a phenolic cap and stirred on a reaction block, preheated to 70 °C, for 12 h. The resulting mixture was cooled to room temperature, extracted with EtOAc, and passed through a short pad of silica gel. The organic extract was concentrated *in vacuo* and purified by flash chromatography over silica gel with Et_2_O/hexanes to obtain pure 4,4′-dimethoxy-1,1′-biphenyl (43.3 mg, 81% yield), the spectral data for which matched that of known material^24^.

### Typical procedure for cross-coupling of 4-iodoacetophenone and 4-iodoanisole

To a sample vial (4 ml) equipped with a Teflon-coated magnetic stir bar were added, in sequence, Pd(OAc)_2_ (25 μmol), magnesium powder (2.5 mmol), 4-iodoacetophenone (0.25 mmol) and 4-iodoanisole (0.50 mmol) (Fig. 3, entry 1). To the mixture was added formaldehyde (37 wt% in water, 1 ml). (**Caution!** The formaldehyde should be added slowly, and the addition should be paused if a vigorous generation of dihydrogen ensues.) The vial was capped and stirred on a reaction block, preheated to 70 °C, for 24 h. The resulting mixture was cooled to room temperature, and then extracted with EtOAc and passed through a short pad of silica gel. The organic extract was concentrated *in vacuo* and purified by flash chromatography over silica gel with Et_2_O/hexanes to obtain pure 1-(4′-methoxy-[1,1′-biphenyl]-4-yl)ethan-1-one (**3a**, 28.8 mg, 51% yield), the spectral data for which matched that of known material^25^.

## Additional information

**How to cite this article:** Bhattacharjya, A. *et al.* Kumada–Grignard-type biaryl couplings on water. *Nat. Commun.* **6**:7401 doi: 10.1038/ncomms8401 (2015).

## Supplementary Material

Supplementary Information Supplementary Figures 1-31, Supplementary Table 1, Supplementary Methods and Supplementary References

## Figures and Tables

**Figure 1:**
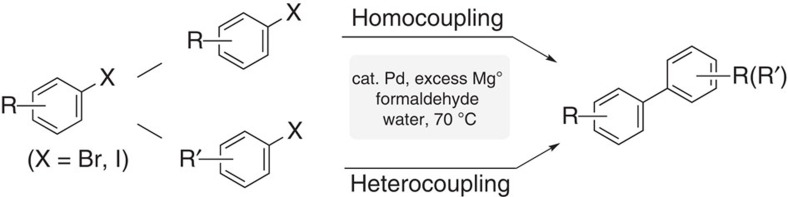
A Kumada-type biaryl coupling in water. Homo- and hetero-coupling with catalytic Pd(II) and excess magnesium.

**Table 1:**