# Supplementary material for: Kumada–Grignard-type biaryl couplings on water
Source: Nat Commun. 2015 Jun 18;6:7401. doi: 10.1038/ncomms8401 (PMC4647940; doi:10.1038/ncomms8401)
Supplement: Supplementary Information — Supplementary Figures 1-31, Supplementary Table 1, Supplementary Methods and Supplementary References [file ncomms8401-s1.pdf]

Supplementary Figures

^{1}H NMR Spectra

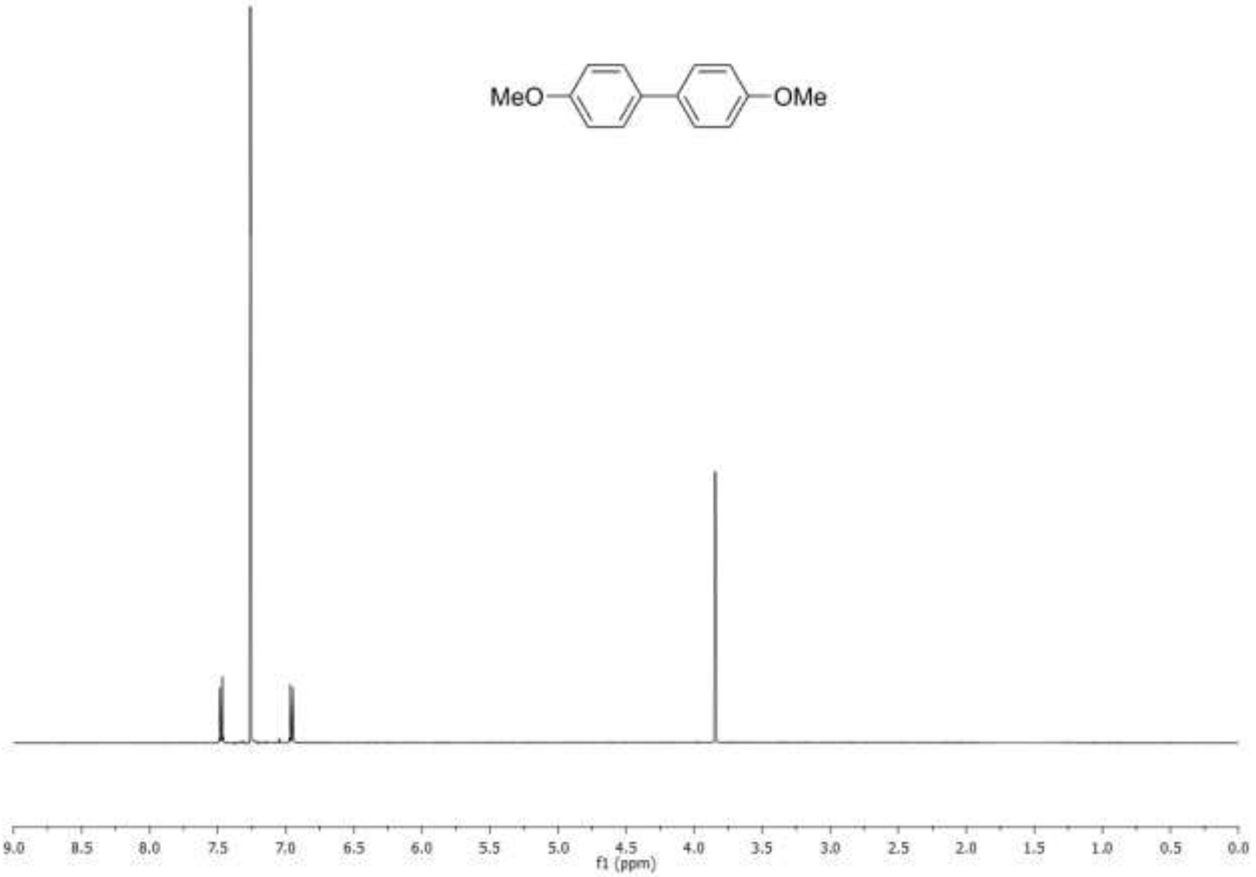

Supplementary Figure 1. ^{1}H NMR Spectrum for Table 2, entry 1

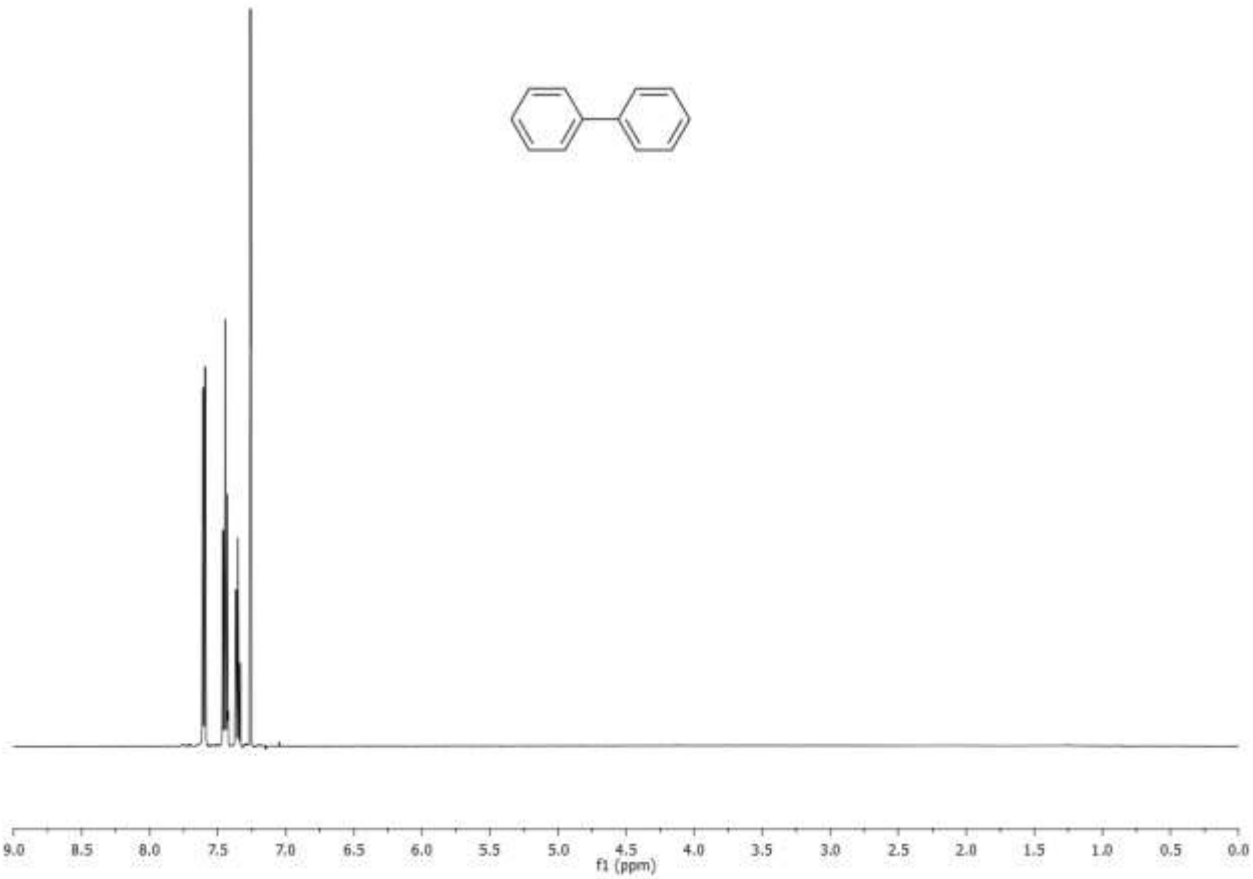

Supplementary Figure 2. ^{1}H NMR Spectrum for Table 2, entry 2

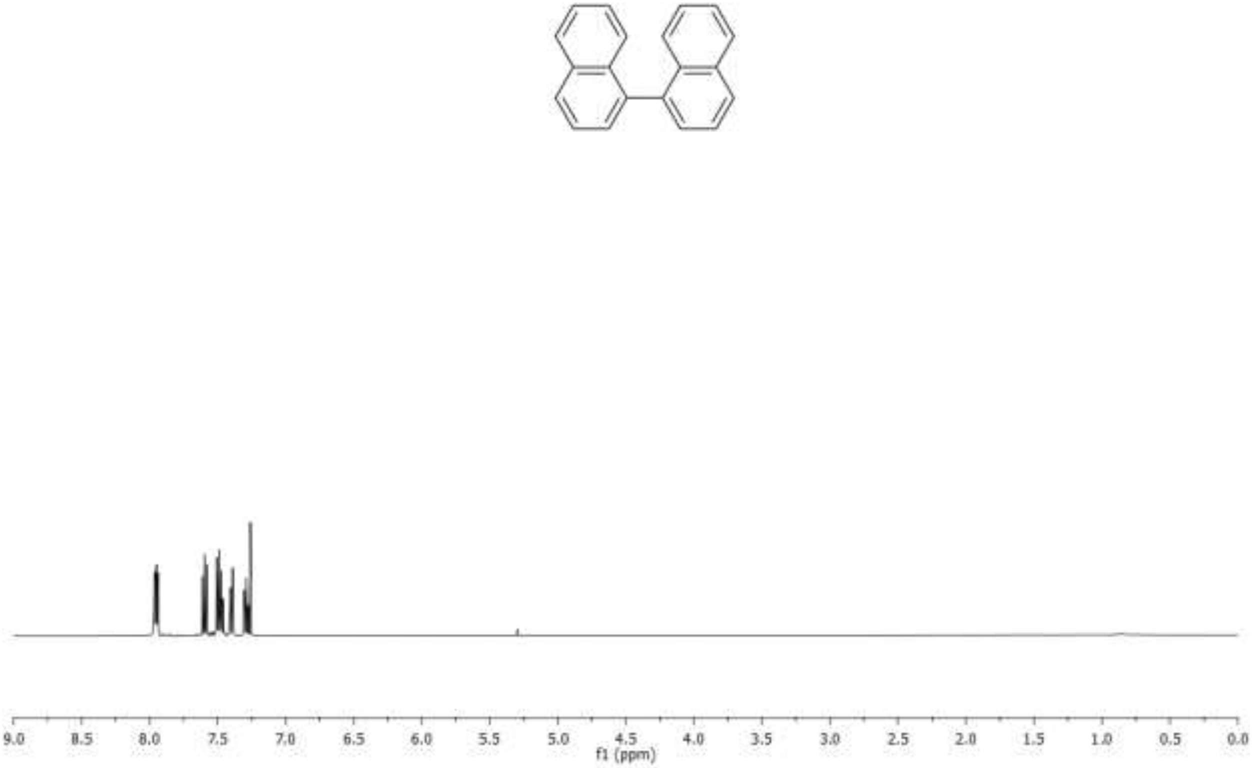

Supplementary Figure 3. ^{1}H NMR Spectrum for Table 2, entry 3

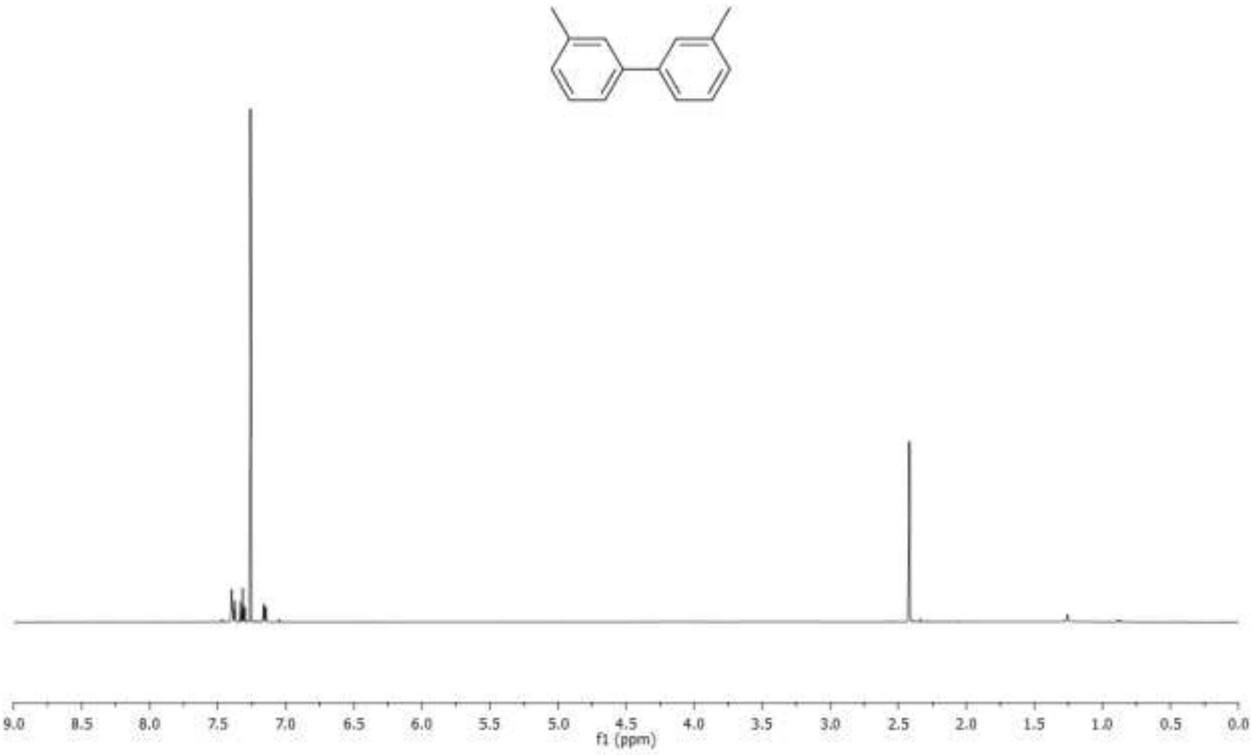

Supplementary Figure 4. ^{1}H NMR Spectrum for Table 2, entry 4

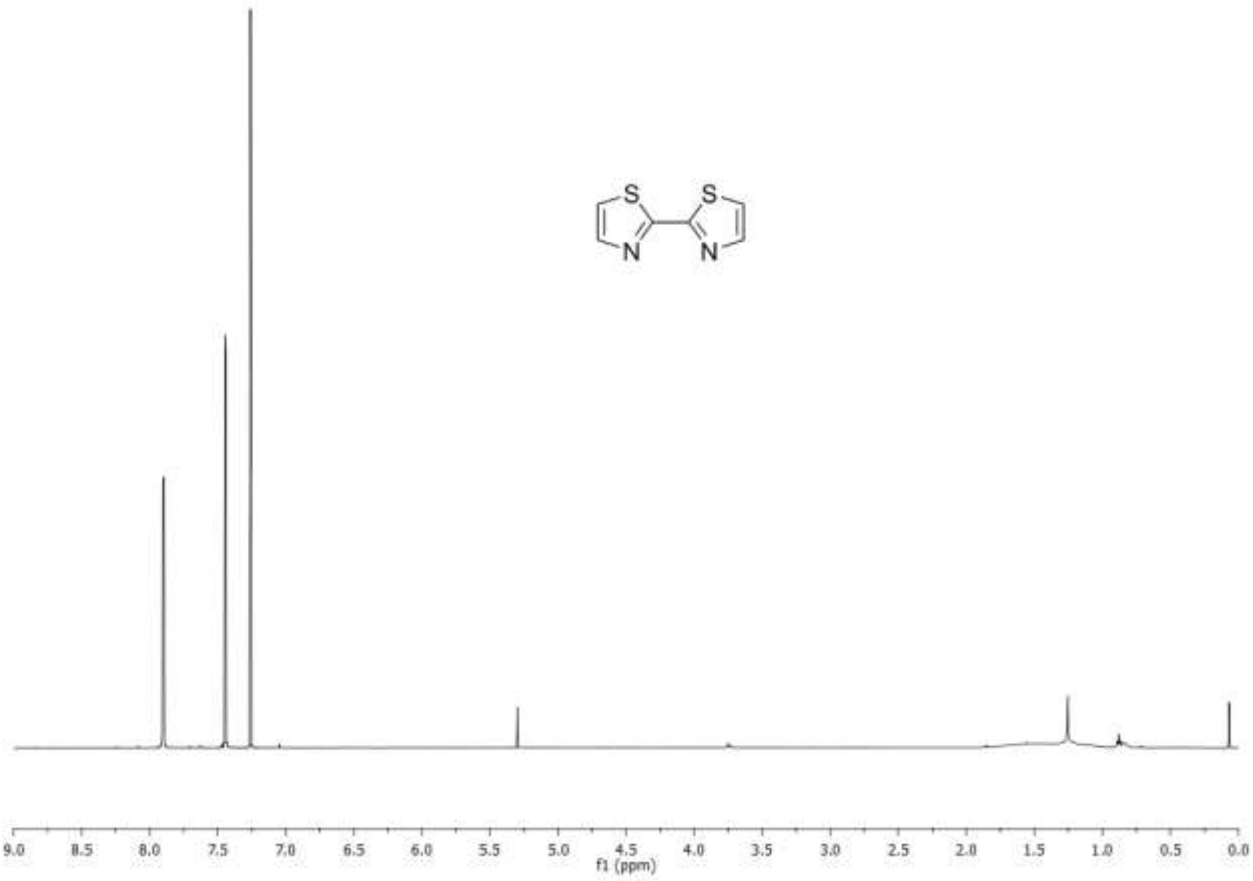

Supplementary Figure 5. ^{1}H NMR Spectrum for Table 2, entry 5

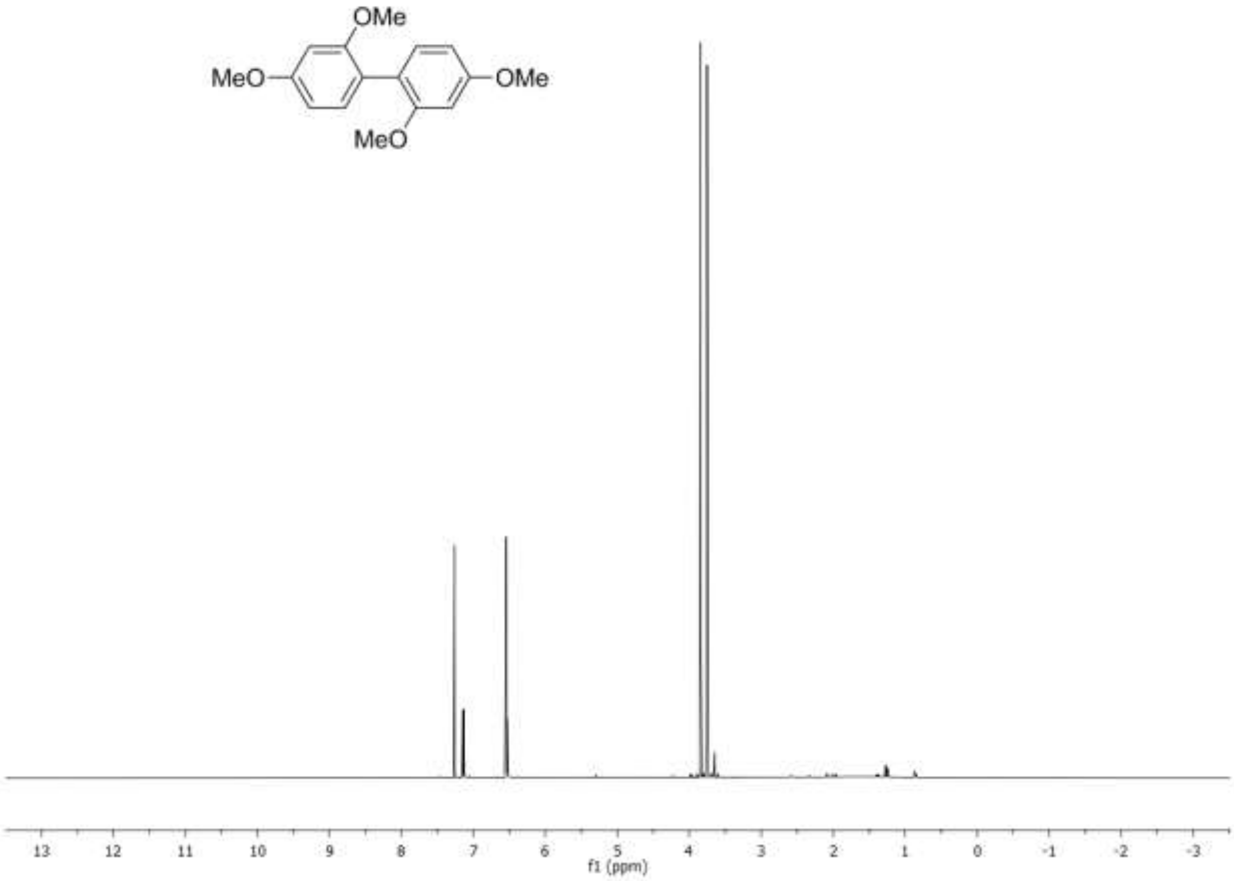

Supplementary Figure 6. ^{1}H NMR Spectrum for Table 2, entry 6

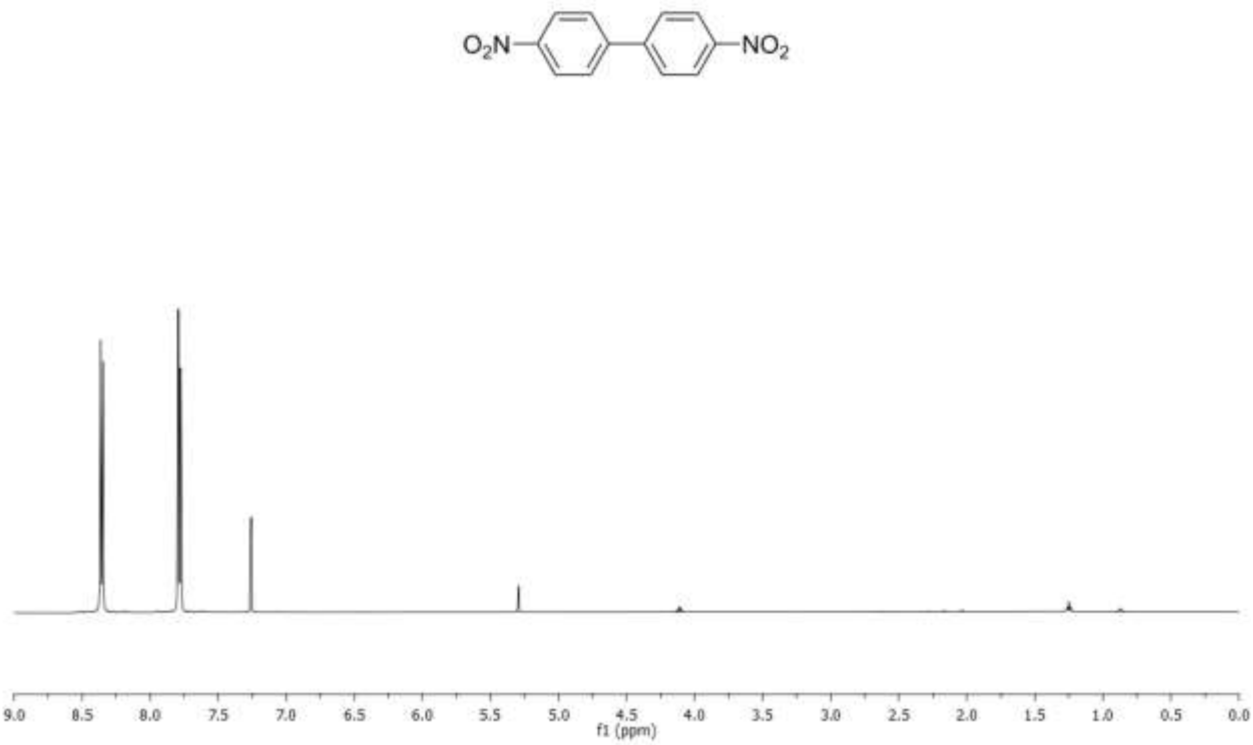

Supplementary Figure 7. ^{1}H NMR Spectrum for Table 2, entry 7

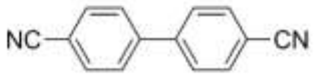

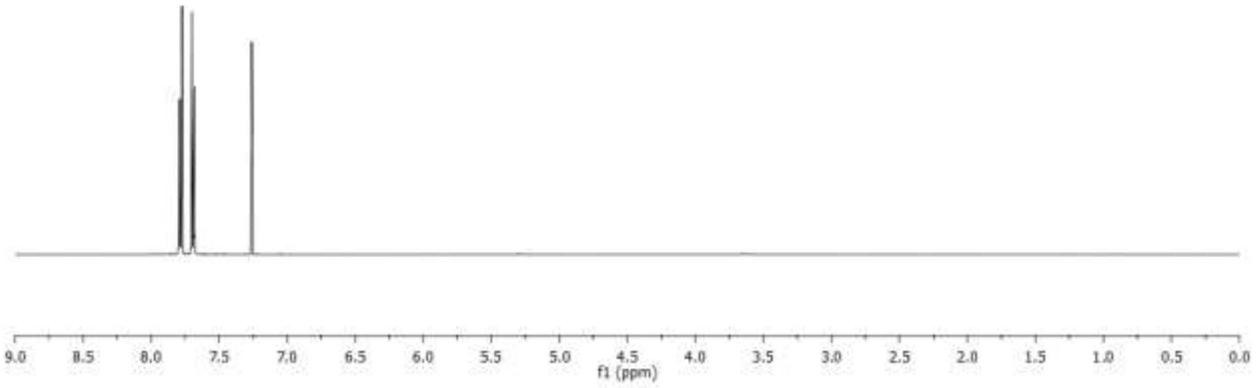

Supplementary Figure 8. ^{1}H NMR Spectrum for Table 2, entry 8

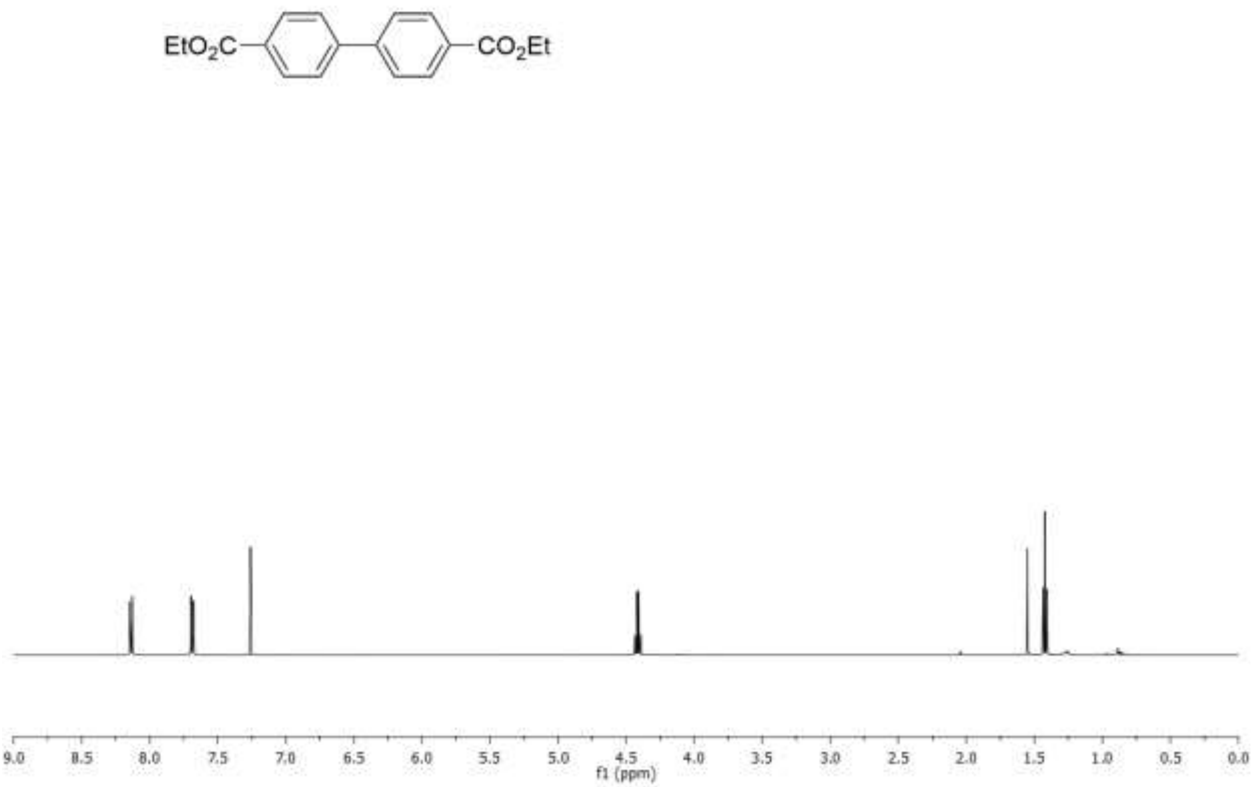

Supplementary Figure 9. ^{1}H NMR Spectrum for Table 2, entry 9

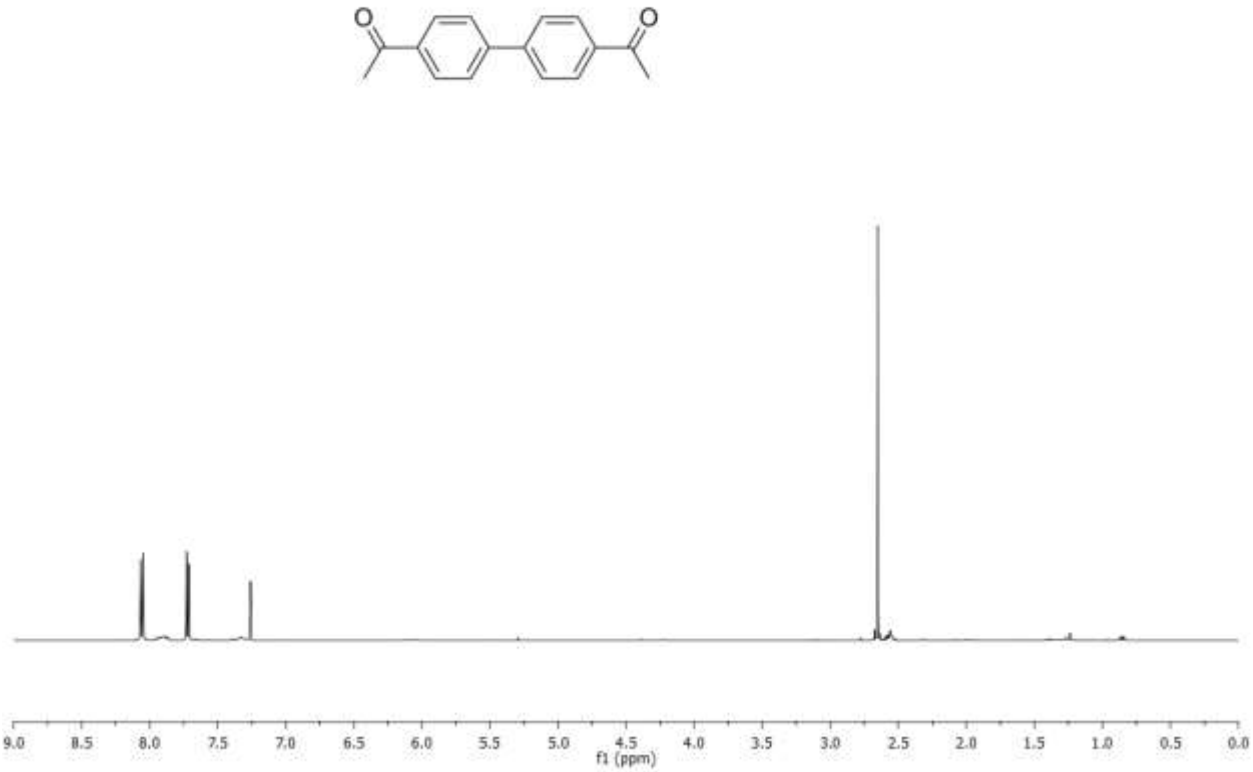

Supplementary Figure 10. ^{1}H NMR Spectrum for Table 2, entry 10

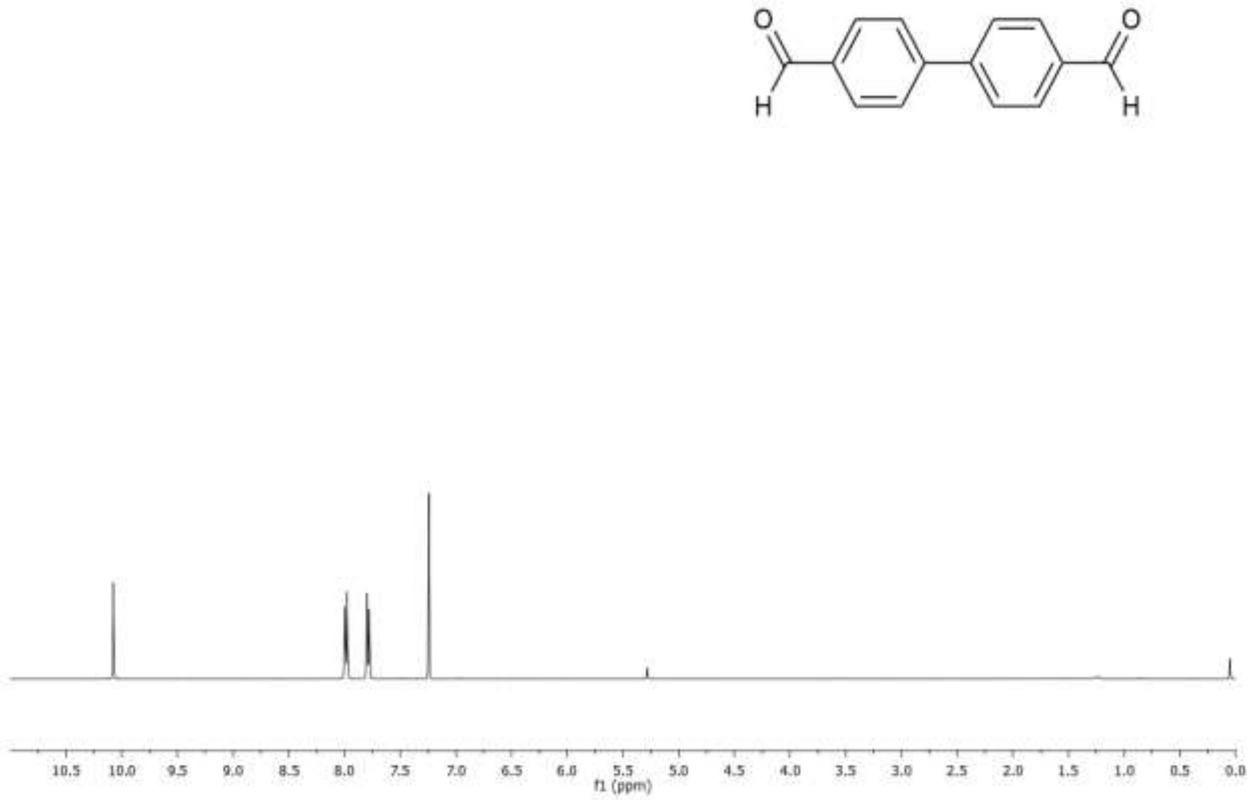

Supplementary Figure 11. ^{1}H NMR Spectrum for Table 2, entry 11

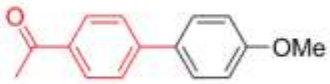

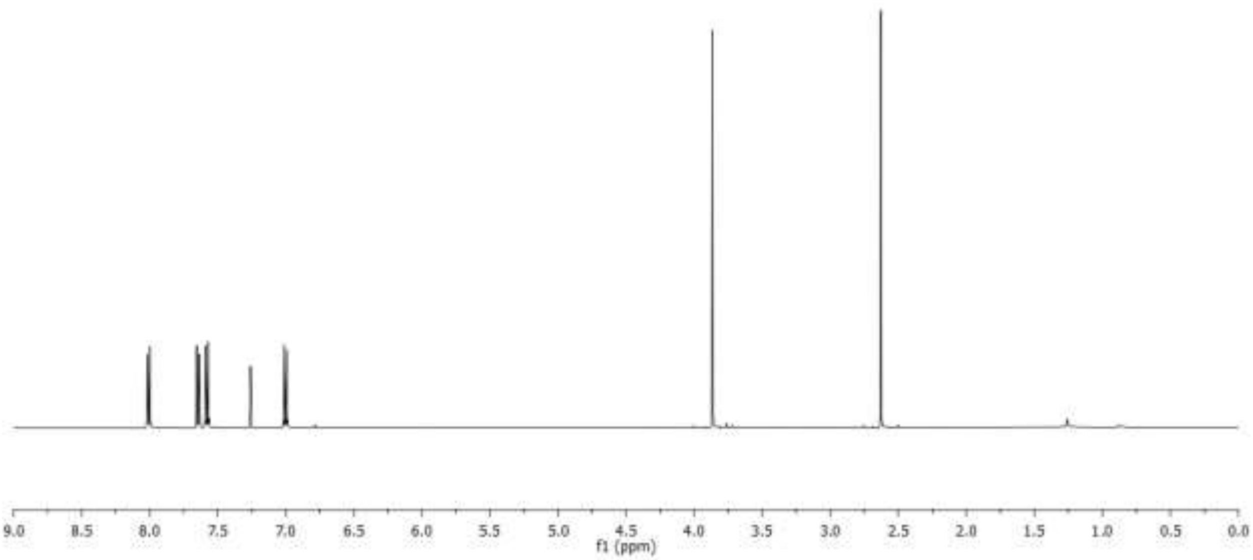

Supplementary Figure 12. ^{1}H NMR Spectrum for Table 3, entry 1

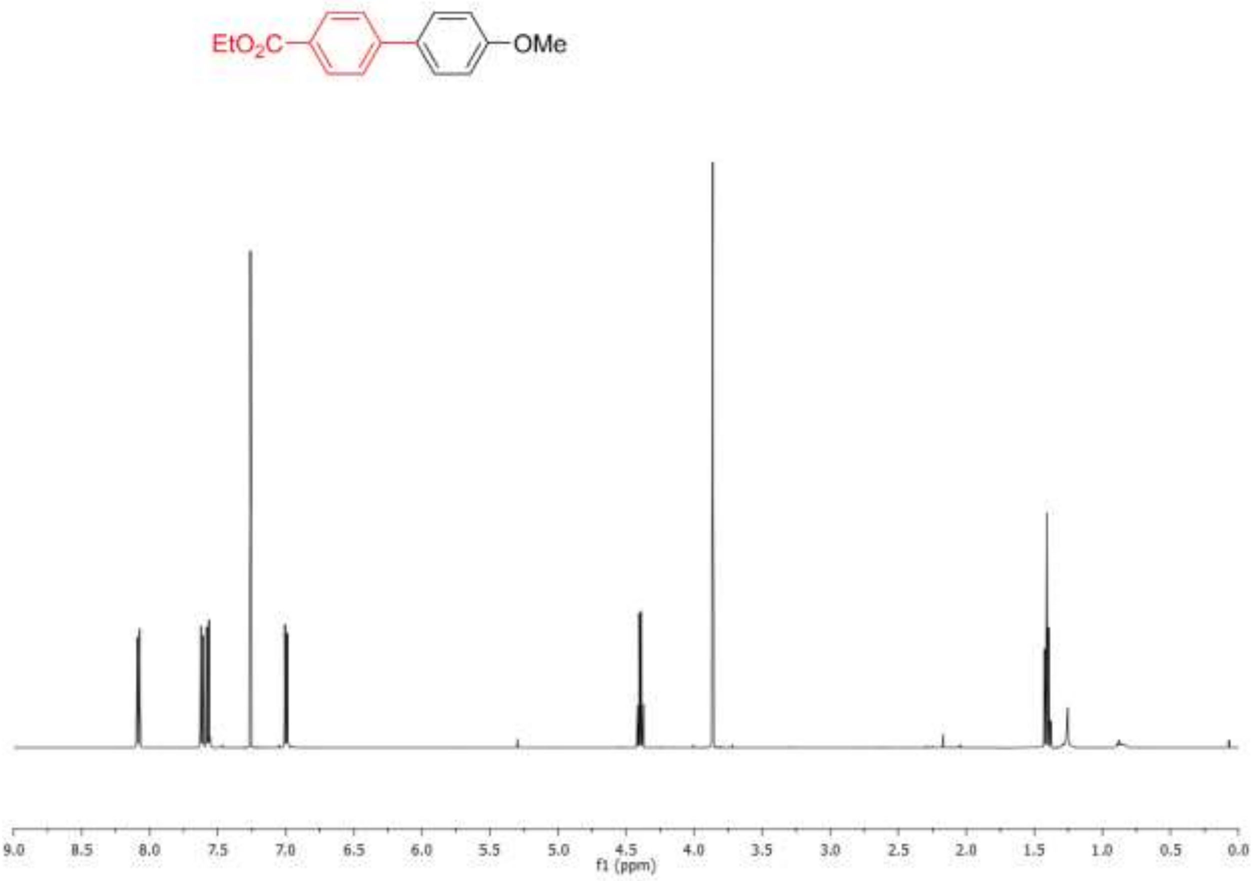

Supplementary Figure 13. ^{1}H NMR Spectrum for Table 3, entry 2

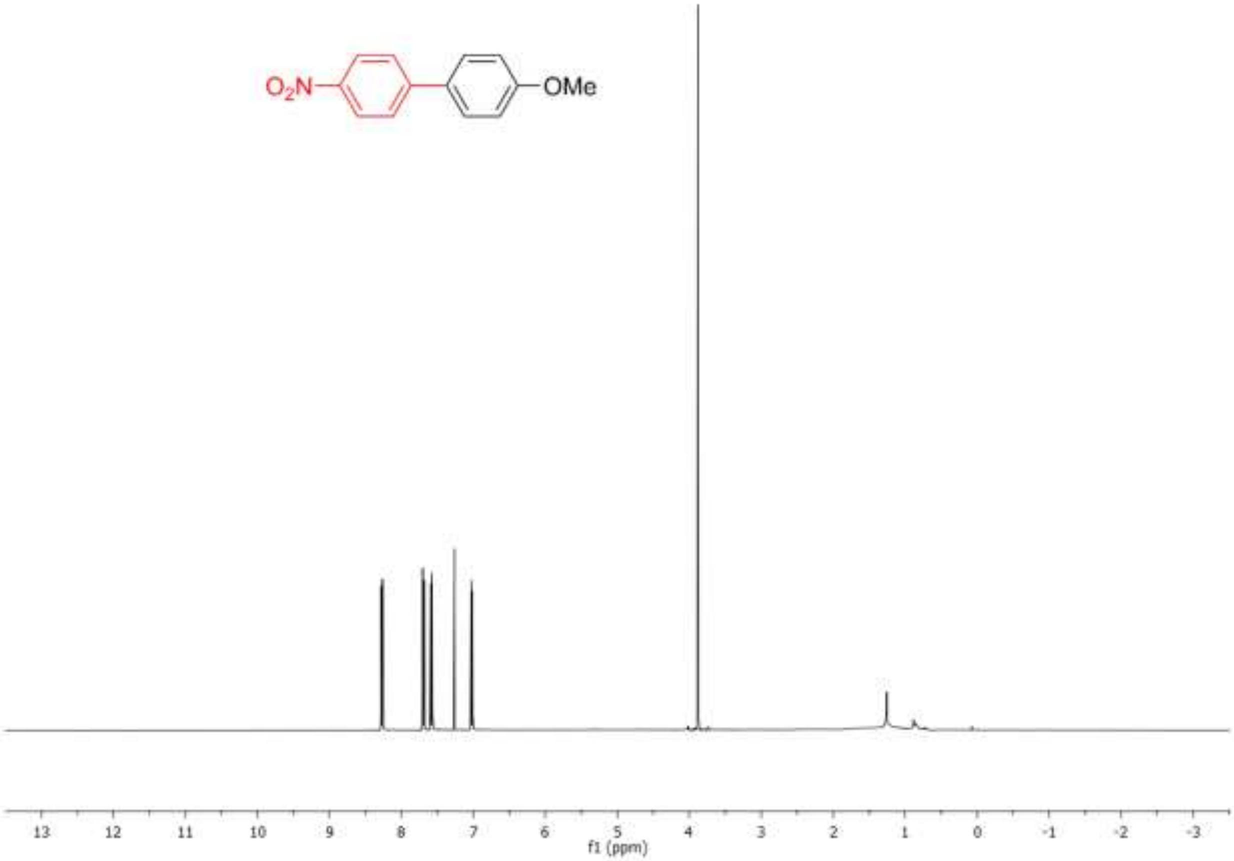

Supplementary Figure 14. ^{1}H NMR Spectrum for Table 3, entry 3

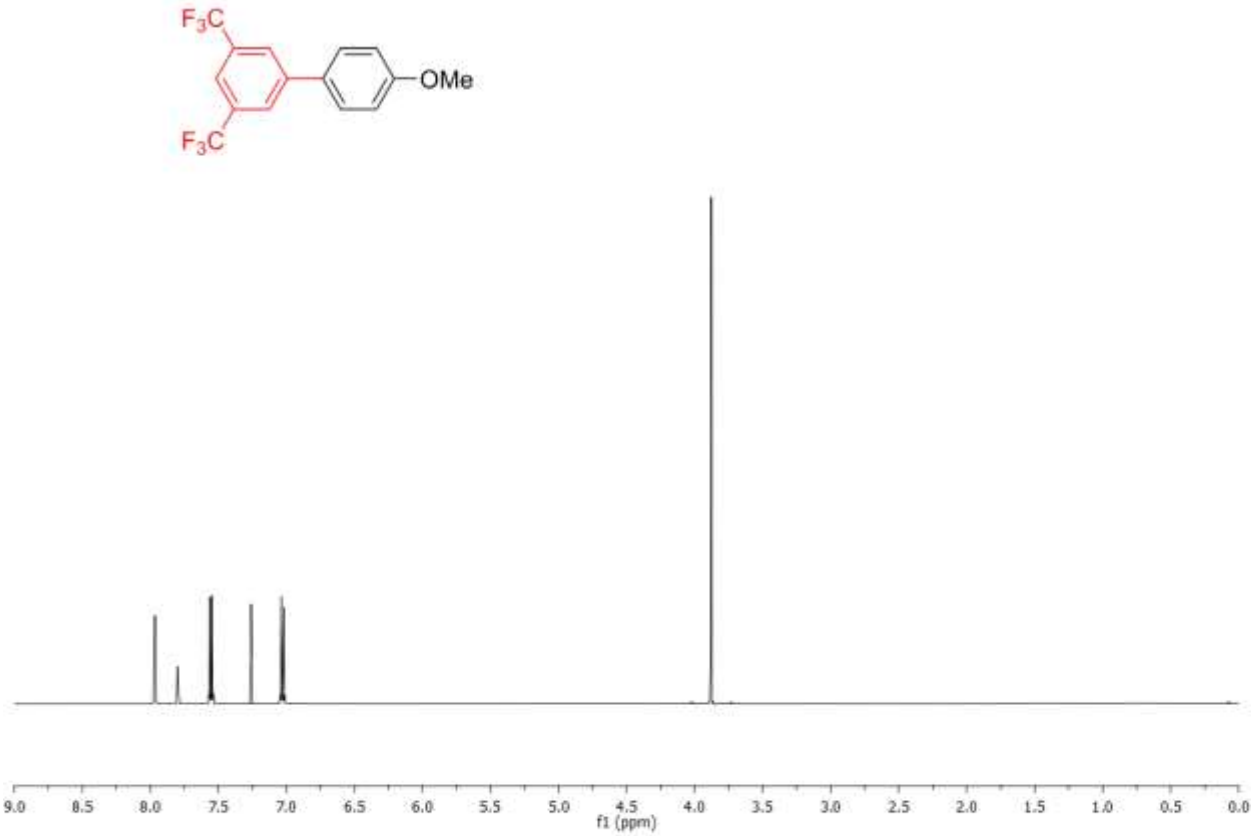

Supplementary Figure 15. ^{1}H NMR Spectrum for Table 3, entry 4

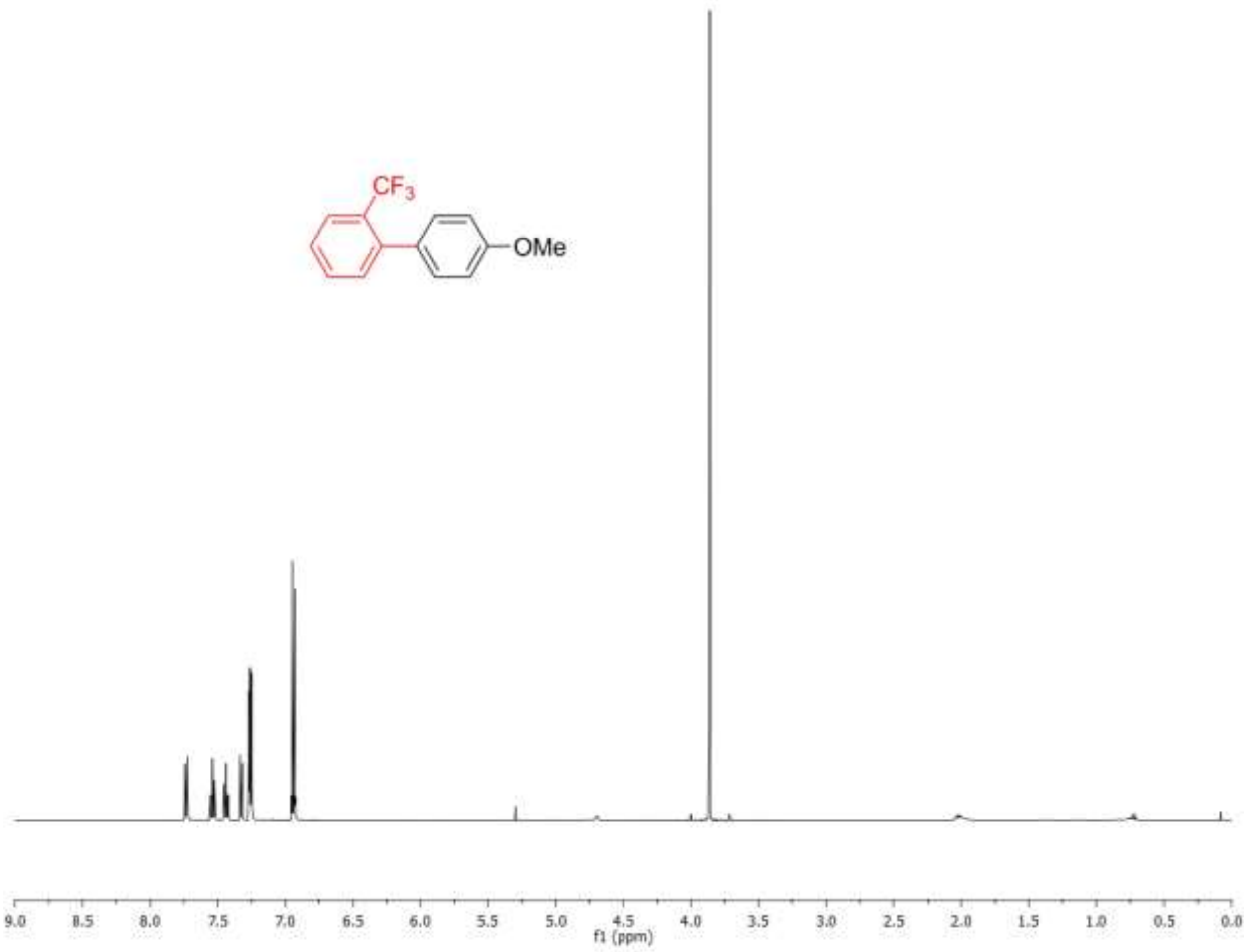

Supplementary Figure 16. ^{1}H NMR Spectrum for Table 3, entry 5

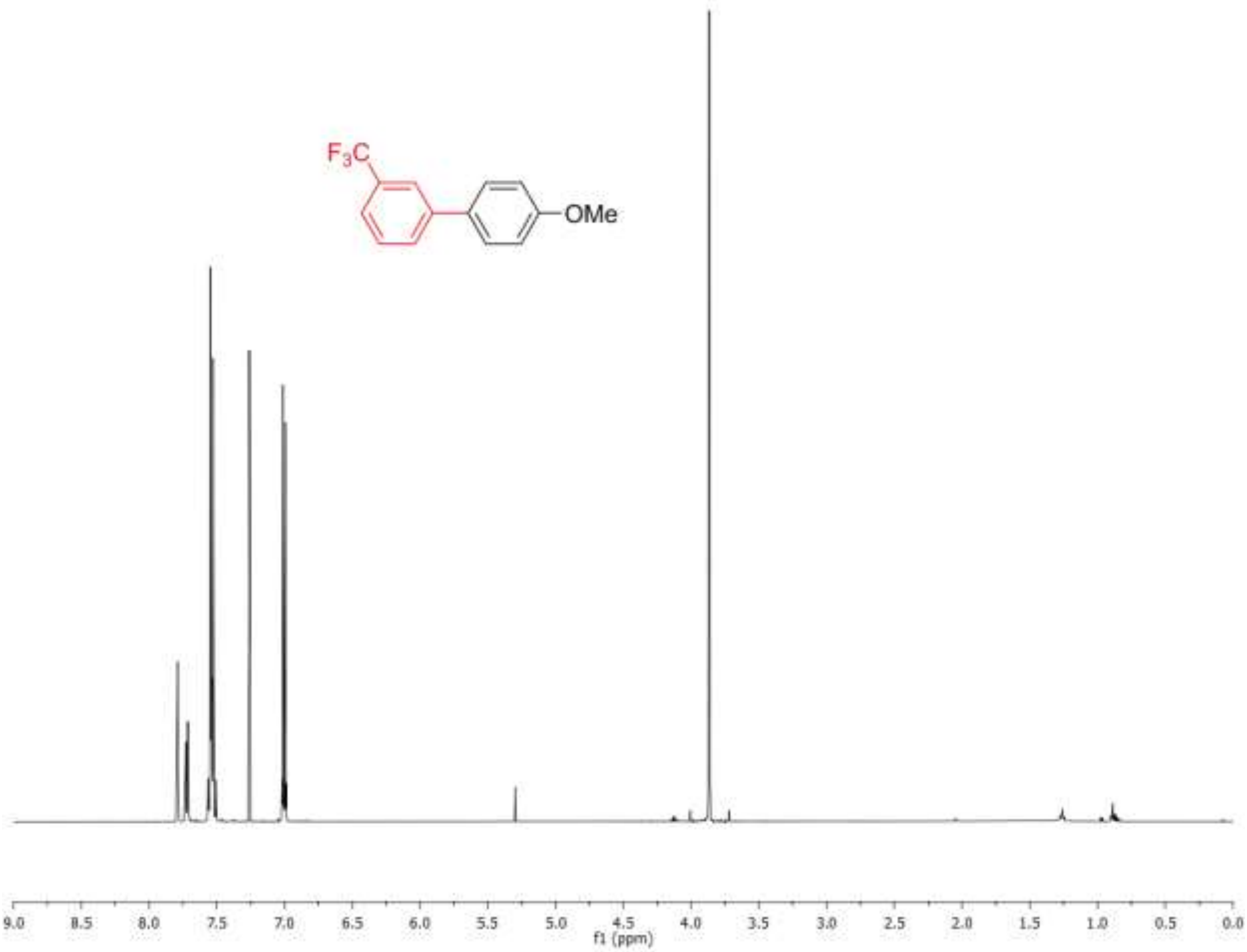

Supplementary Figure 17. ^{1}H NMR Spectrum for Table 3, entry 6

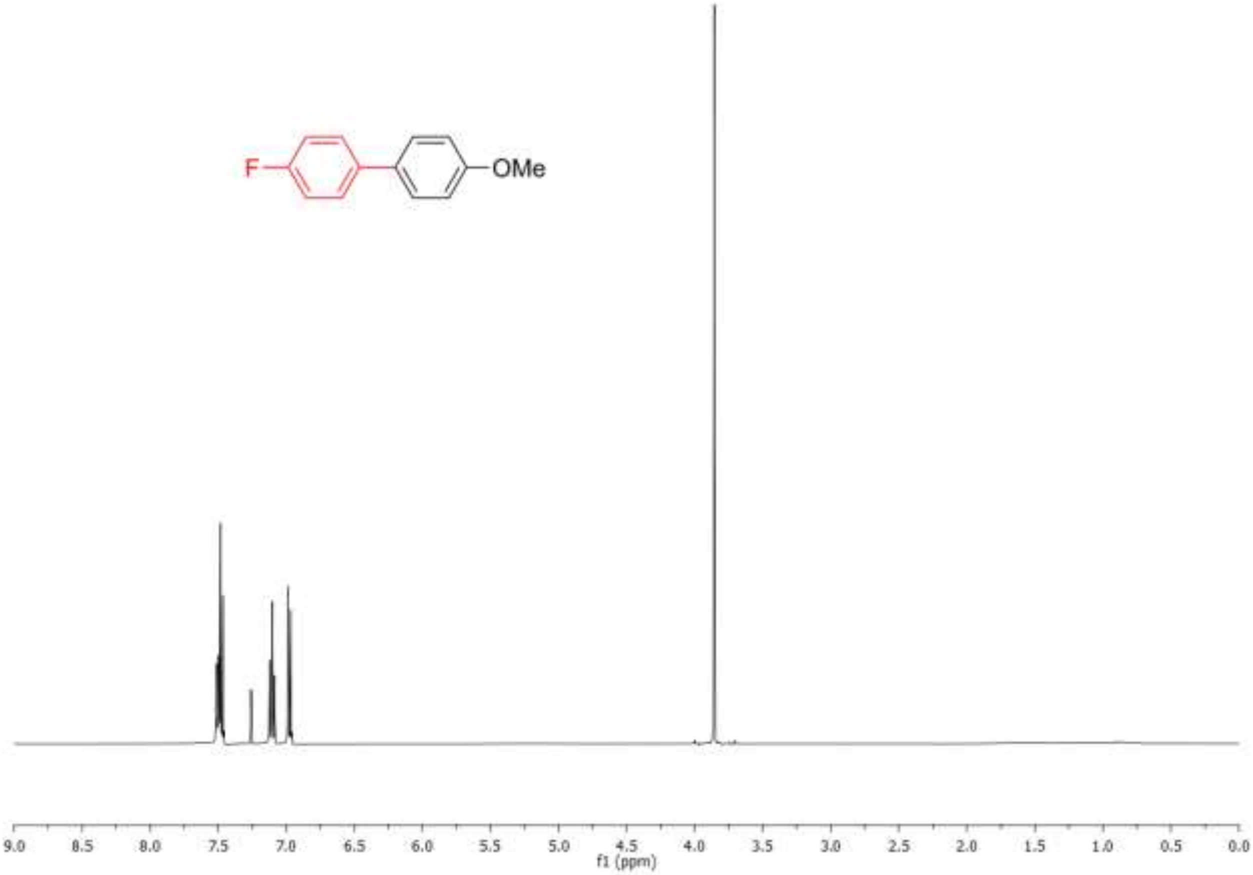

Supplementary Figure 18. ^{1}H NMR Spectrum for Table 3, entry 7

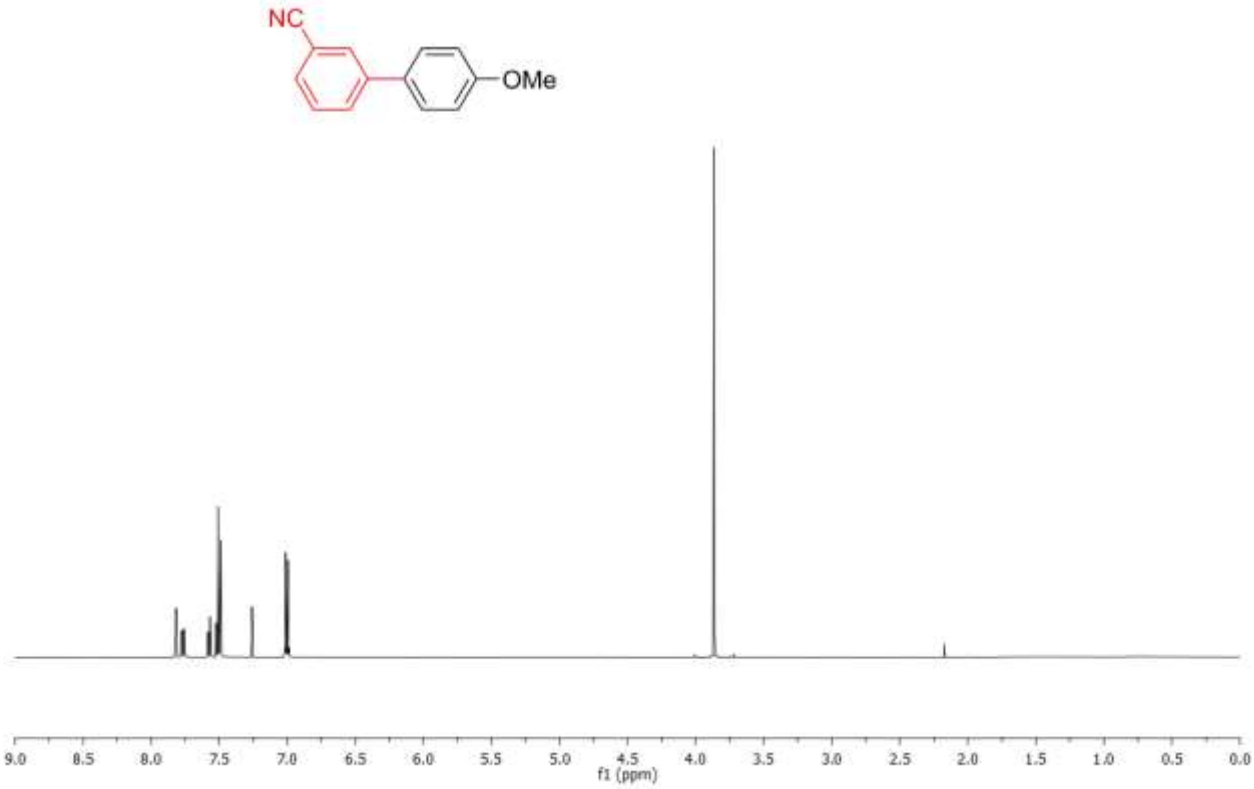

Supplementary Figure 19. ^{1}H NMR Spectrum for Table 3, entry 8

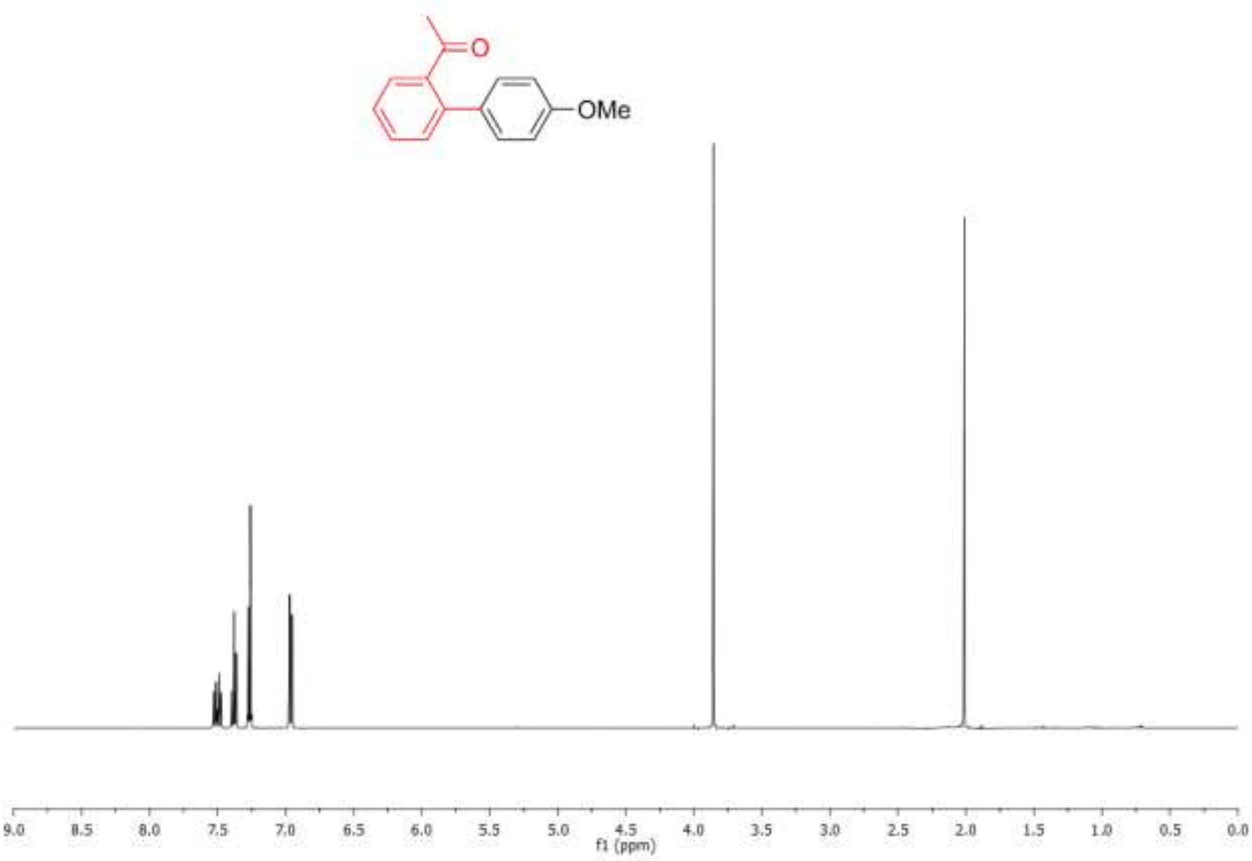

Supplementary Figure 20. ^{1}H NMR Spectrum for Table 3, entry 9

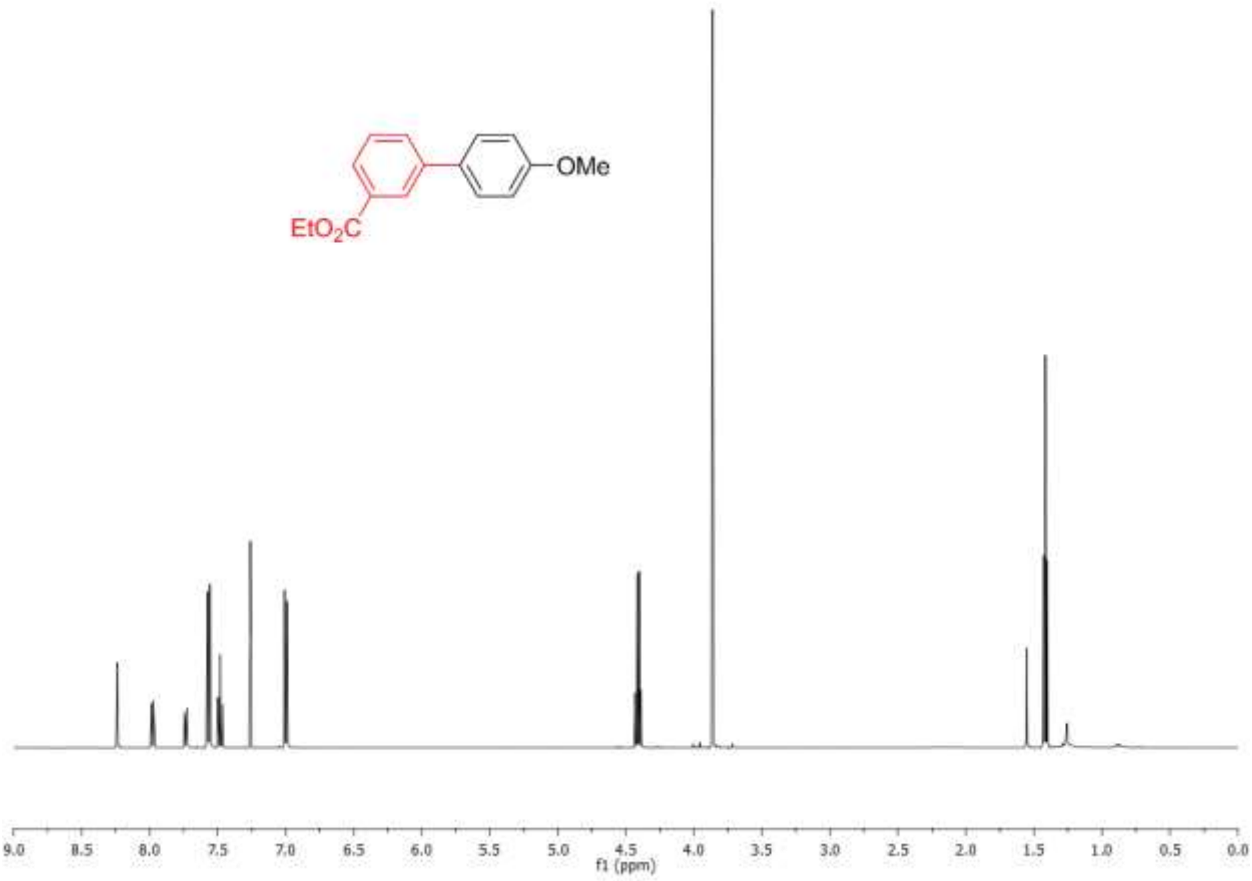

Supplementary Figure 21. ^{1}H NMR Spectrum for Table 3, entry 10

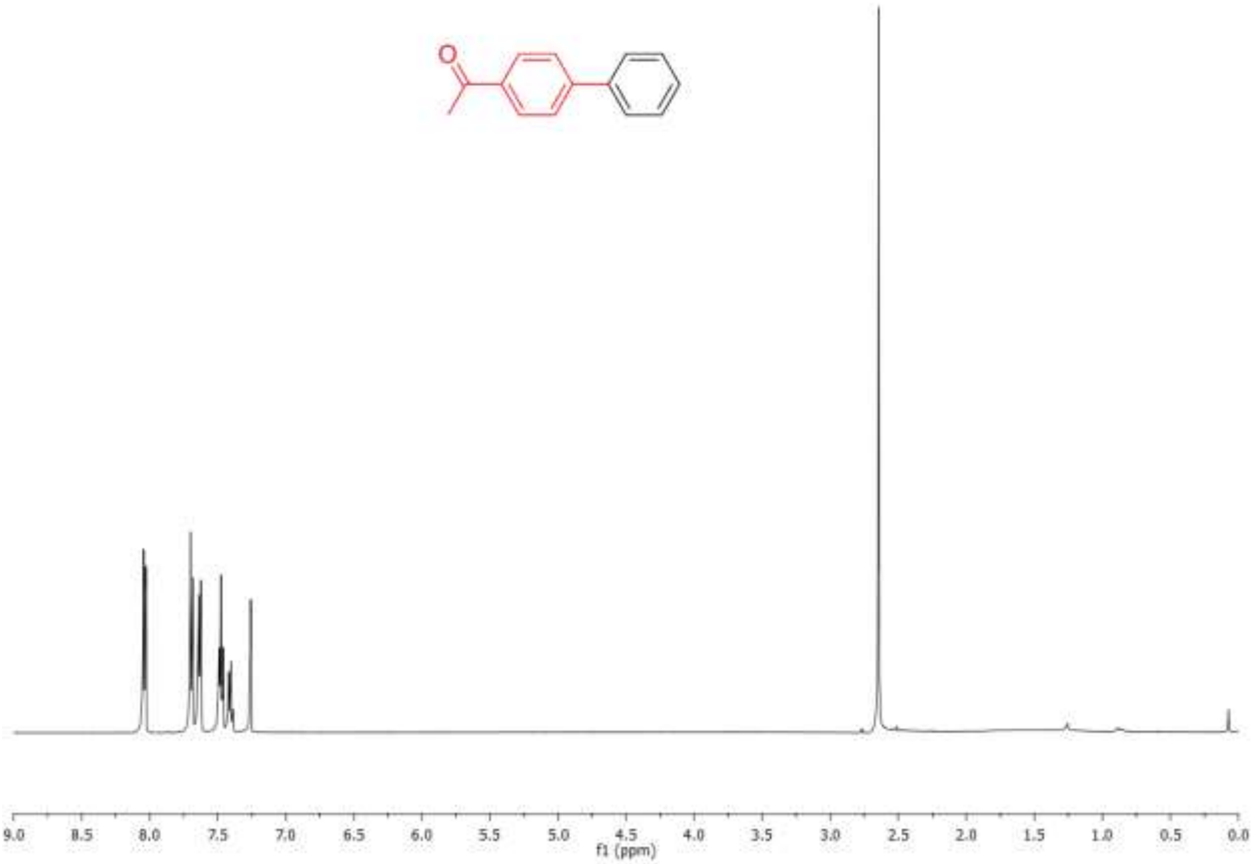

Supplementary Figure 22. ^{1}H NMR Spectrum for Table 3, entry 11

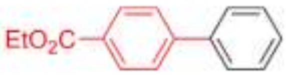

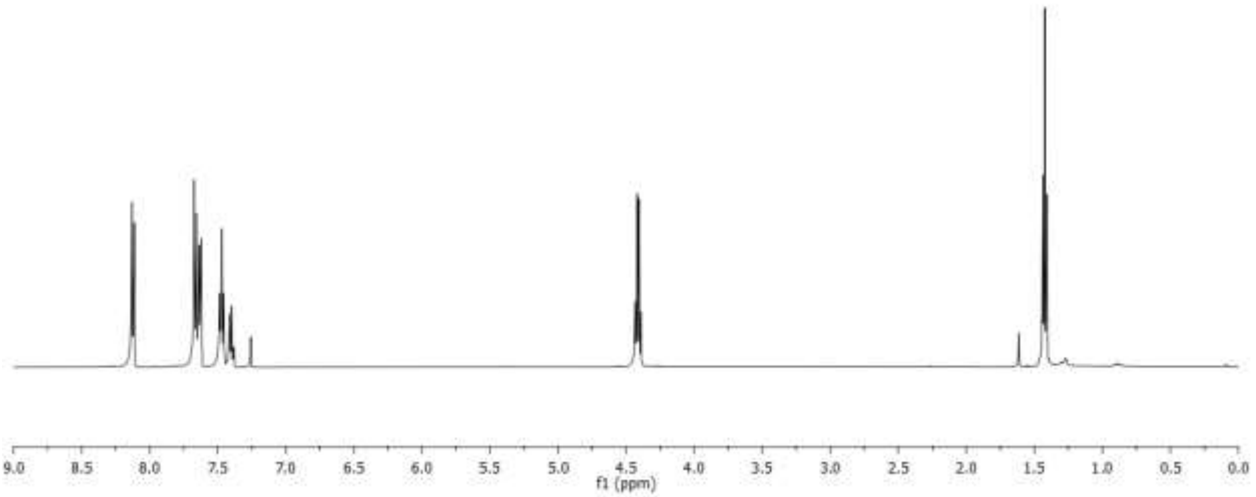

Supplementary Figure 23. ^{1}H NMR Spectrum for Table 3, entry 12

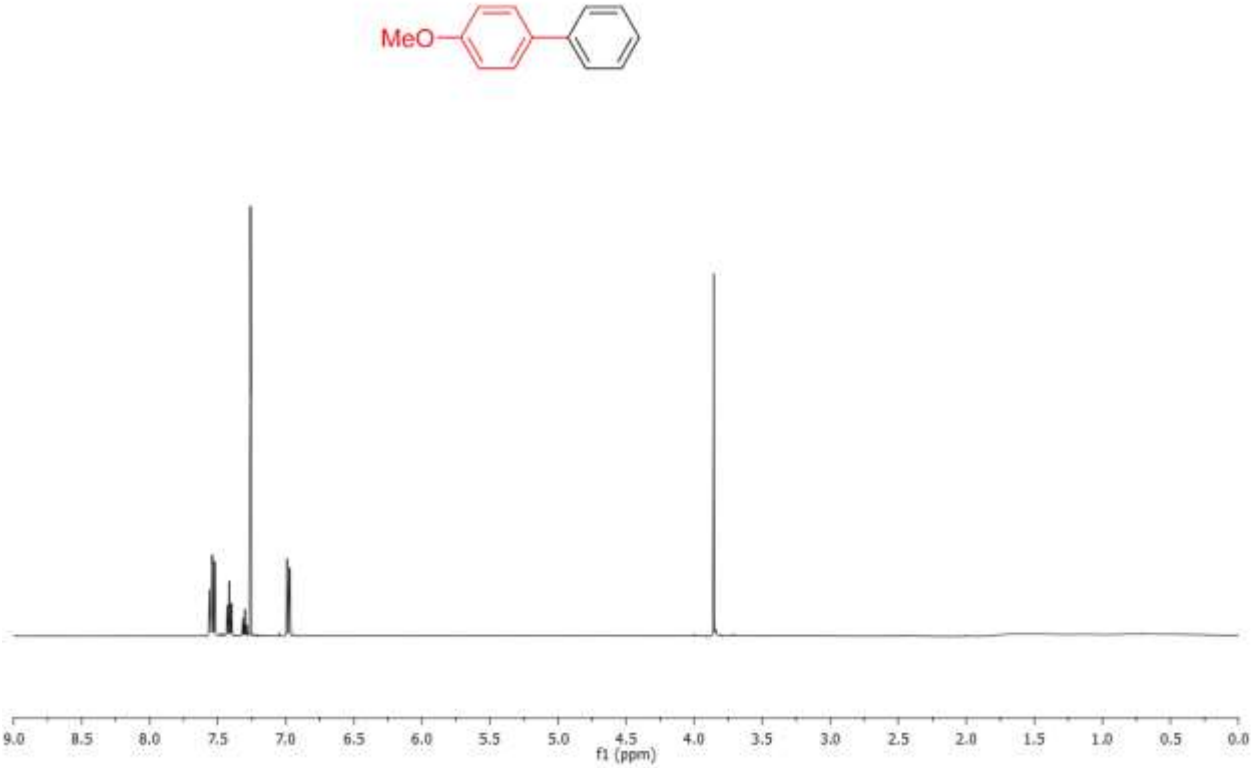

Supplementary Figure 24. ^{1}H NMR Spectrum for Table 3, entry 13

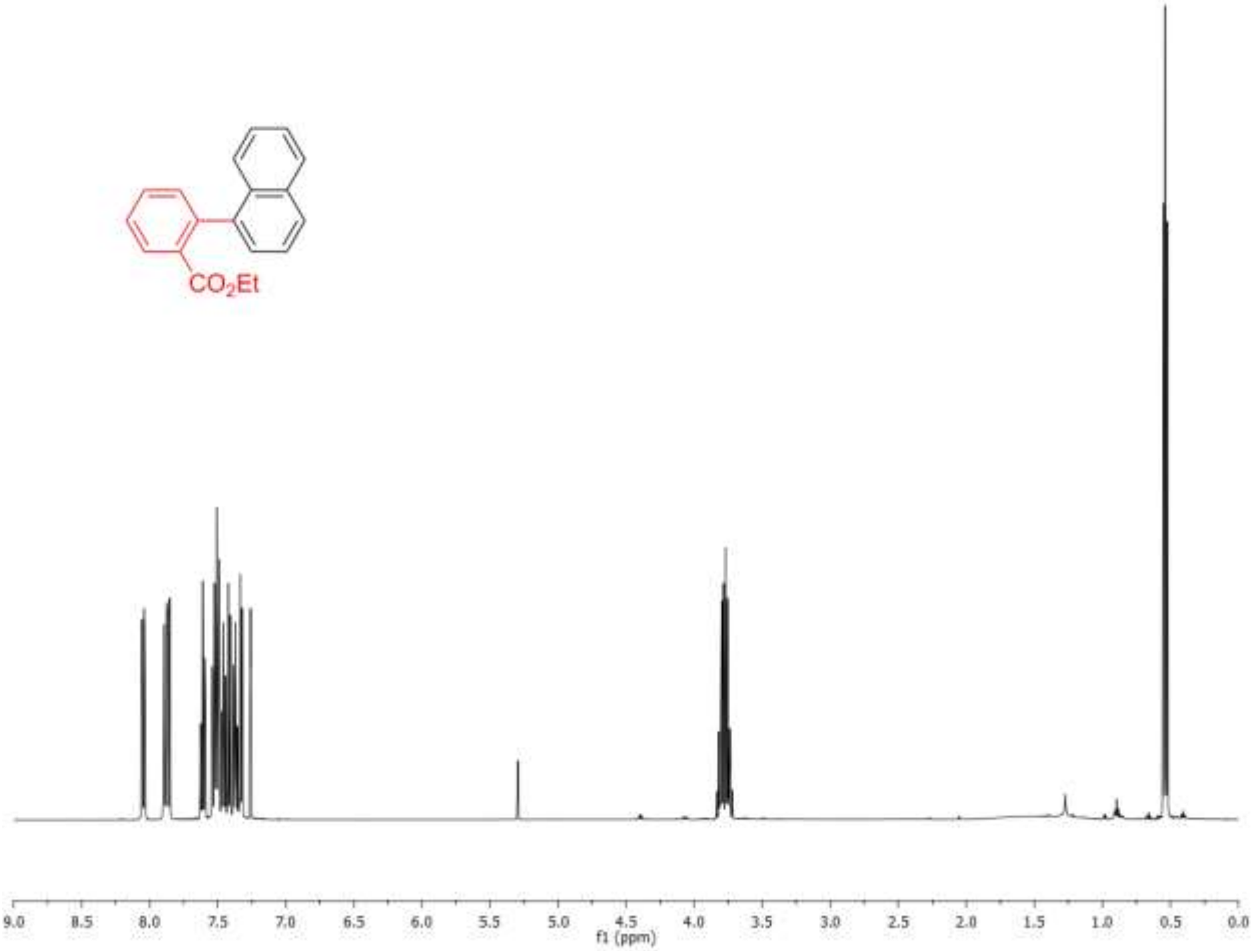

Supplementary Figure 25. ^{1}H NMR Spectrum for Table 3, entry 14

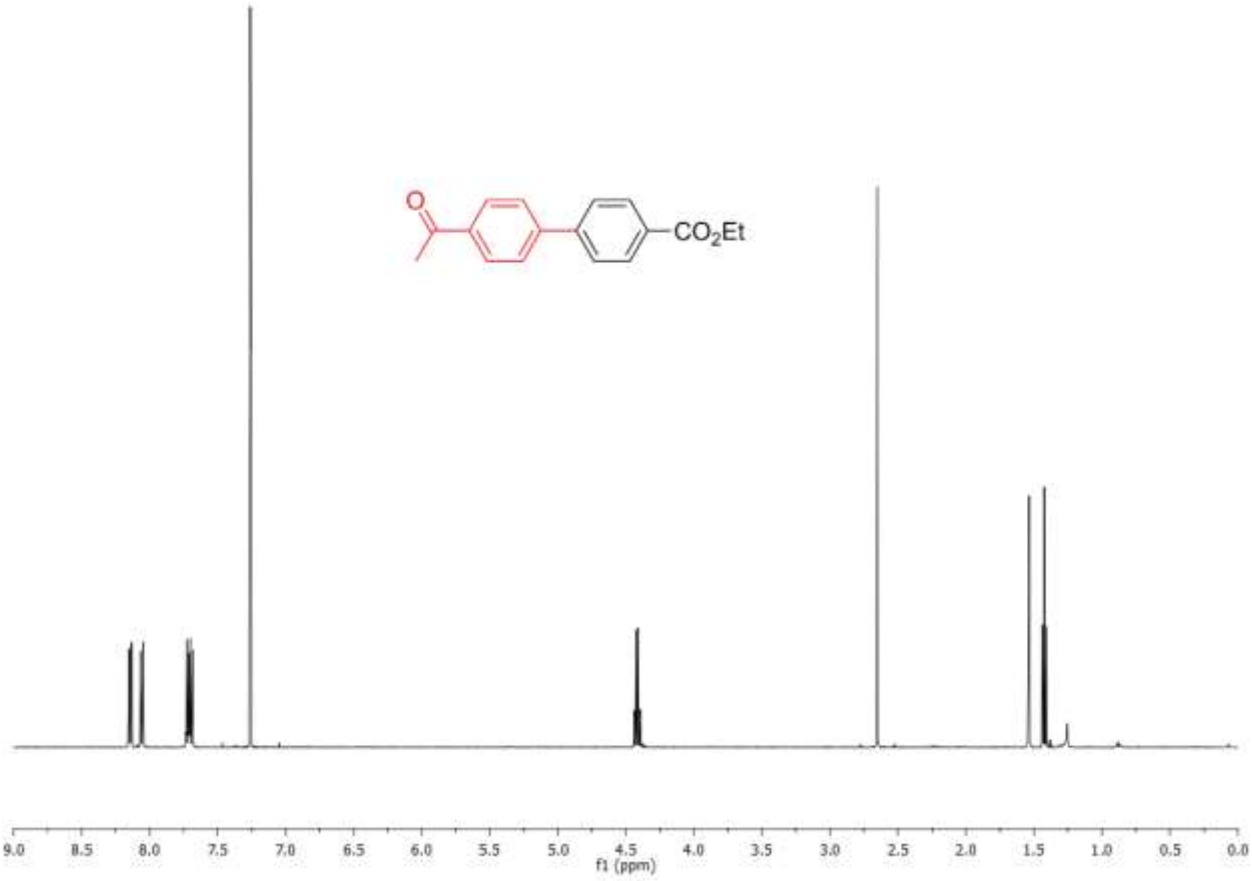

Supplementary Figure 26. ^{1}H NMR Spectrum for Table 3, entry 15

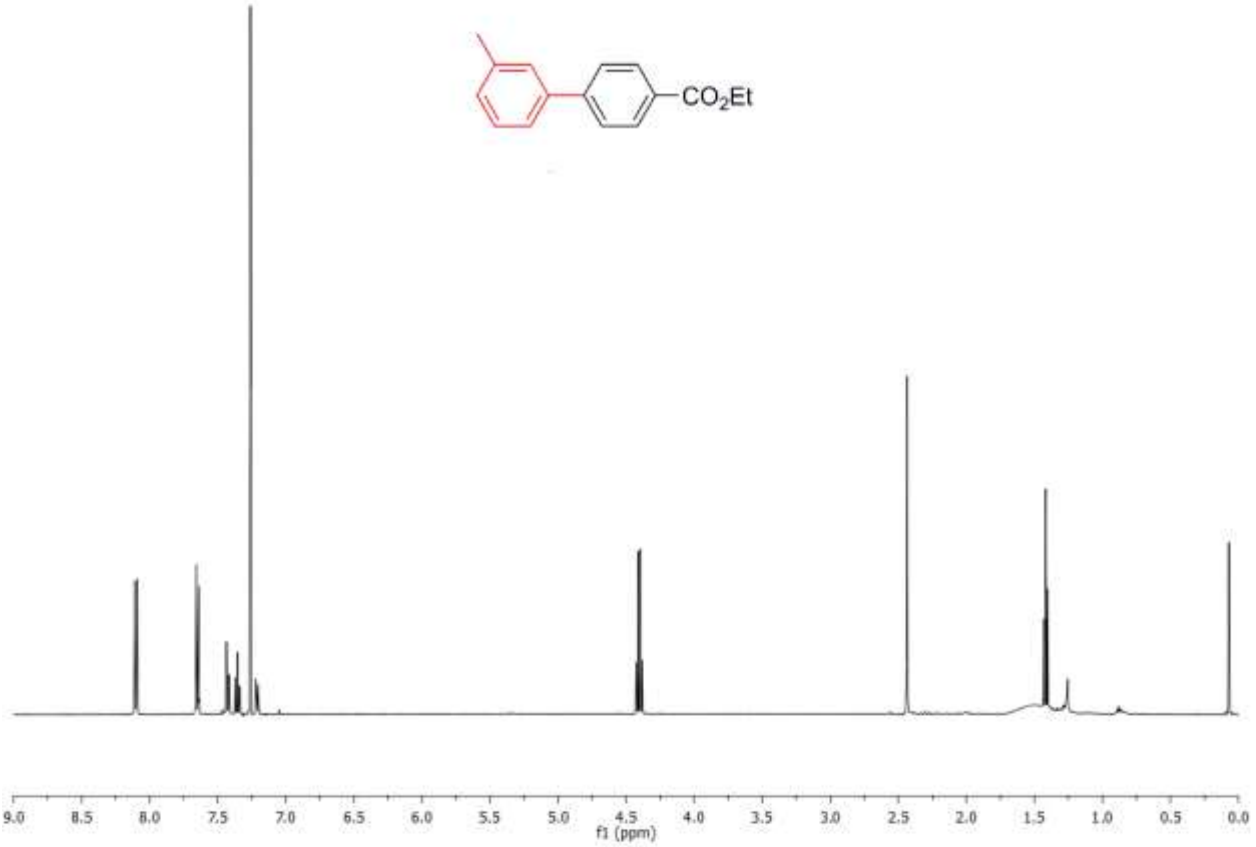

Supplementary Figure 27. ^{1}H NMR Spectrum for Table 3, entry 16

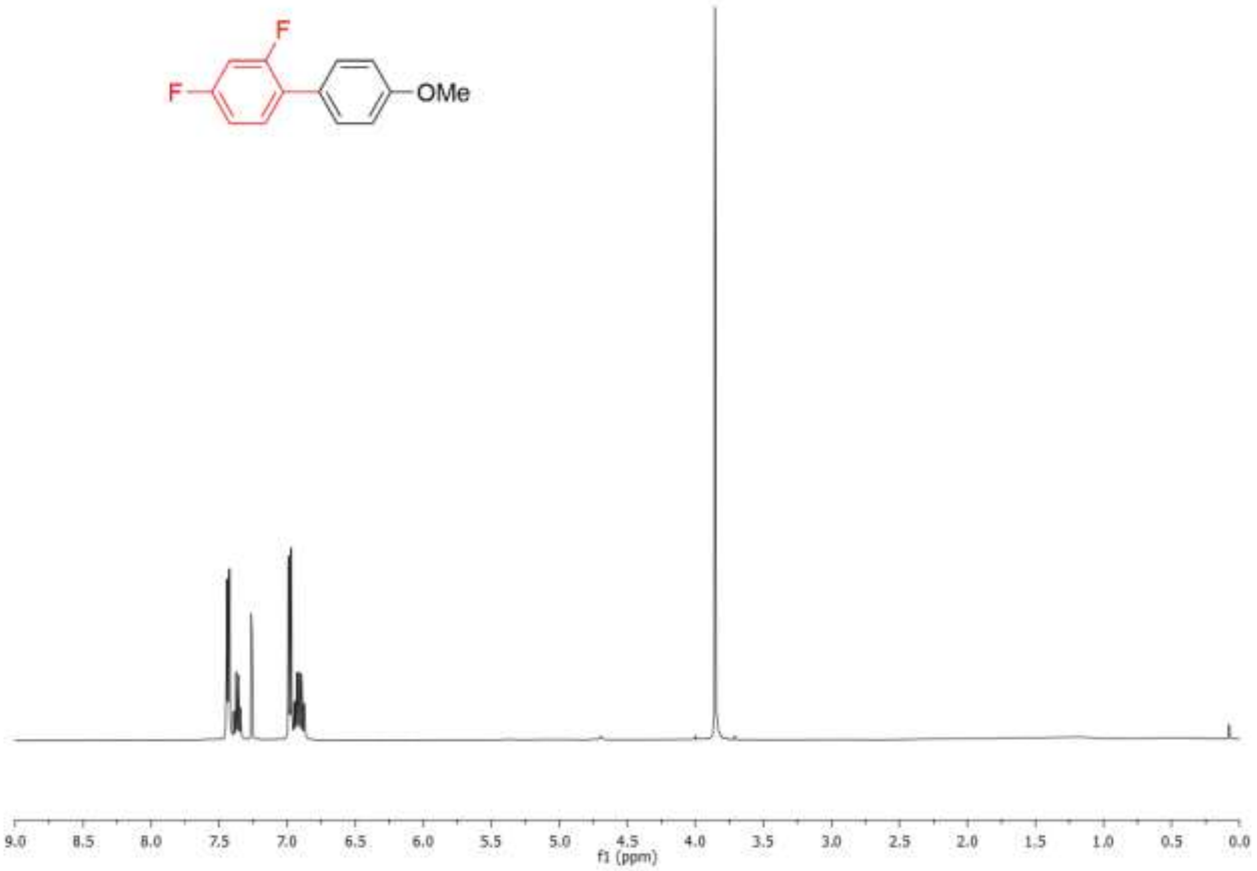

Supplementary Figure 28. ^{1}H NMR Spectrum for Table 3, entry 17

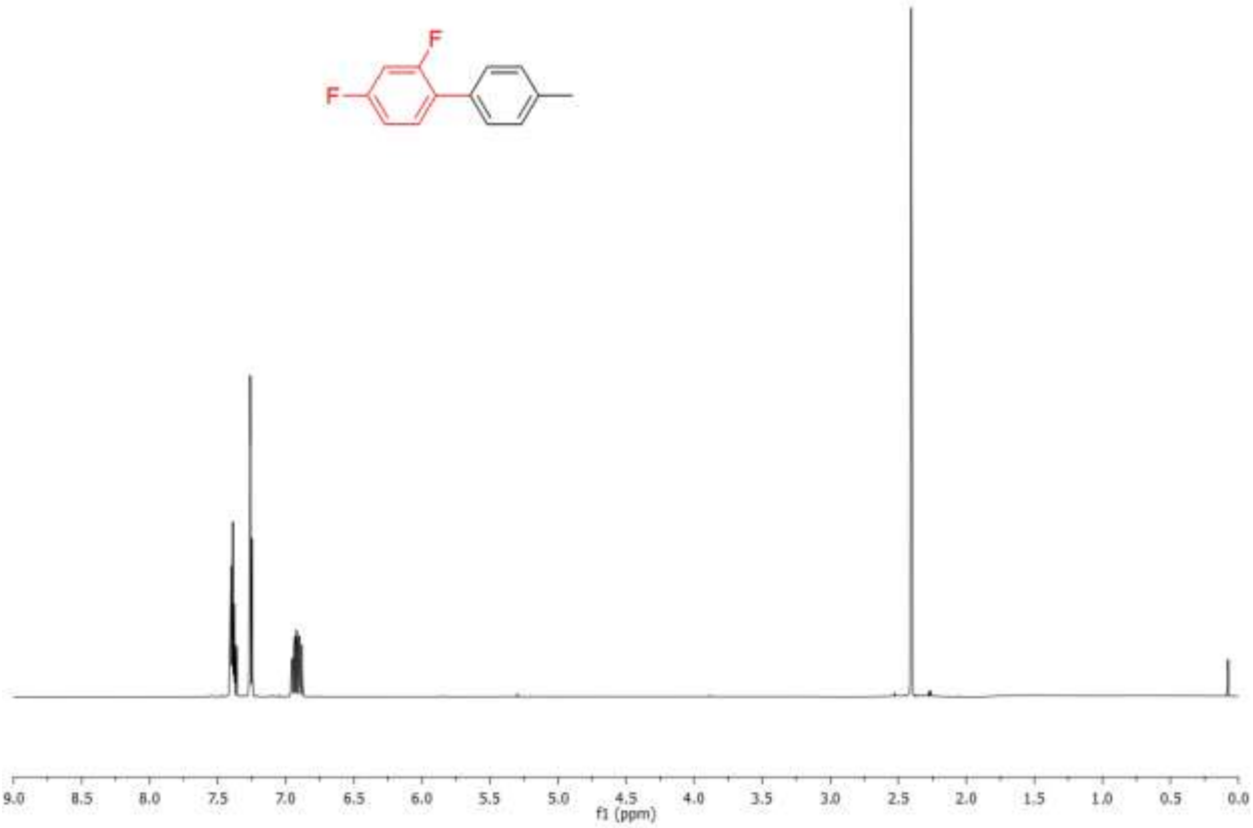

Supplementary Figure 29. ^{1}H NMR Spectrum for Table 3, entry 18

XPS Data

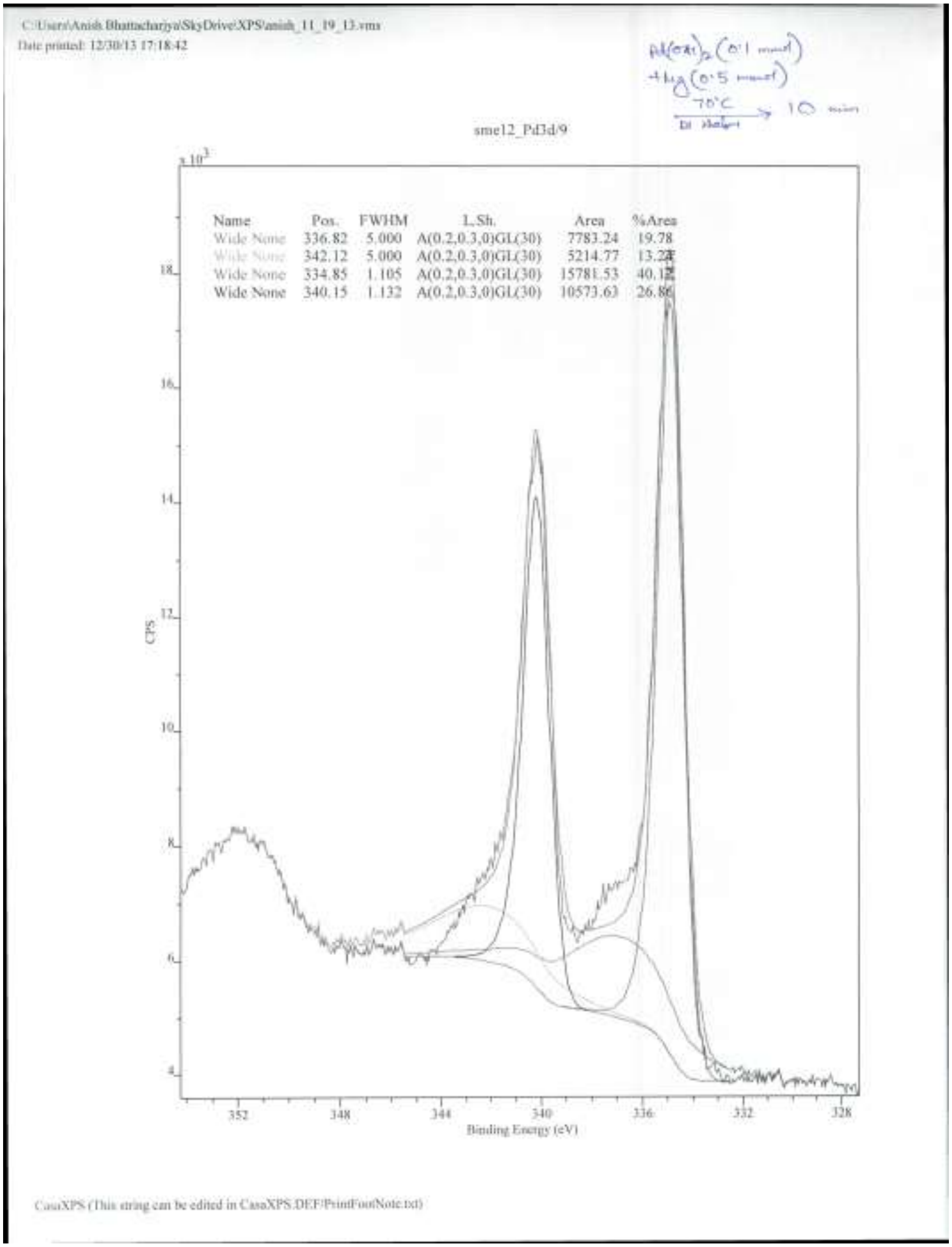

Supplementary Figure 30. X-Ray Photoelectron Spectrum for Pd particles obtained by treating $Pd(OAc)_2$ with Mg in DI water at 70 °C

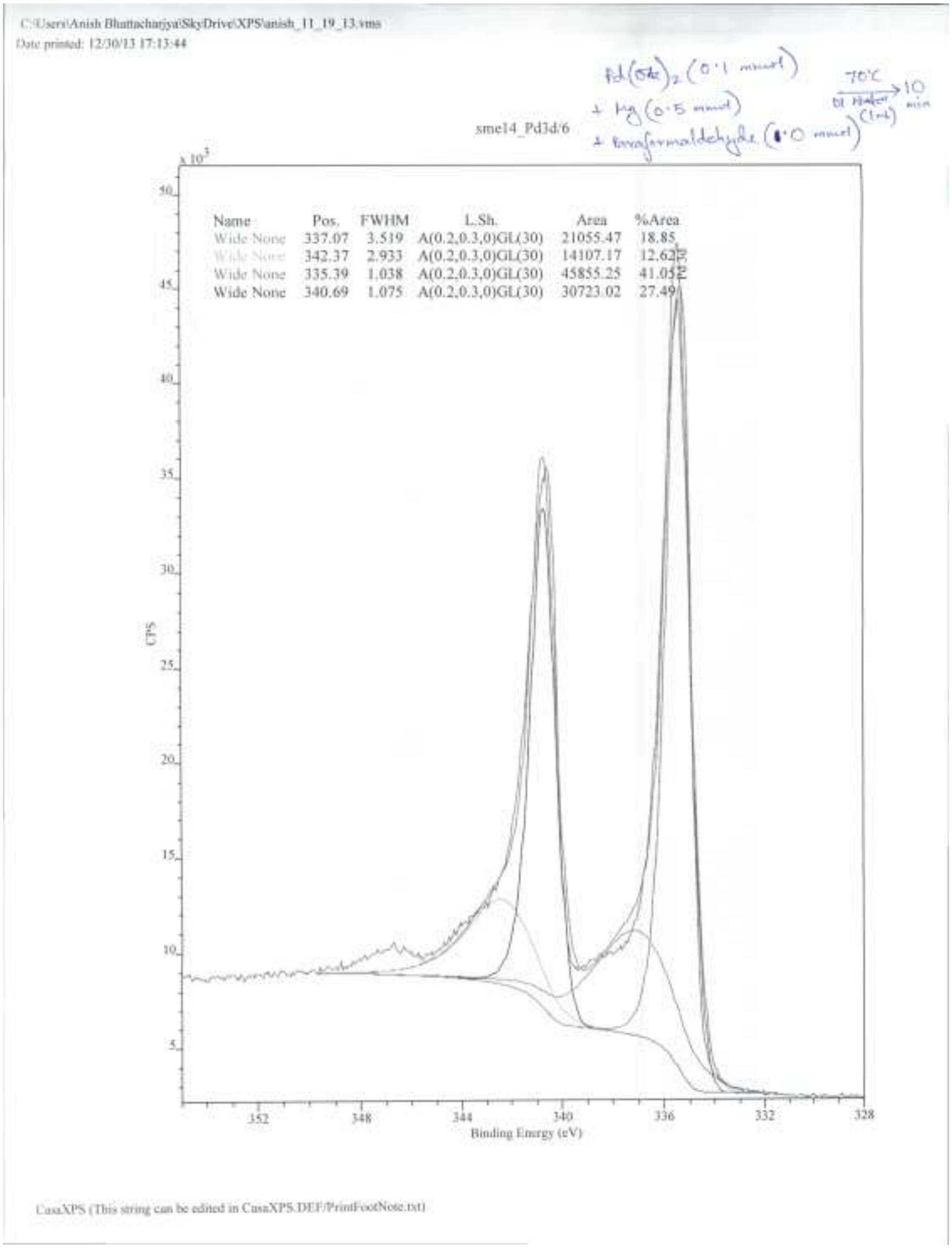

Supplementary Figure 31. X-Ray Photoelectron Spectrum for Pd particles obtained by treating $Pd(OAc)_2$ with Mg and paraformaldehyde in DI water at 70 °C

Supplementary Tables

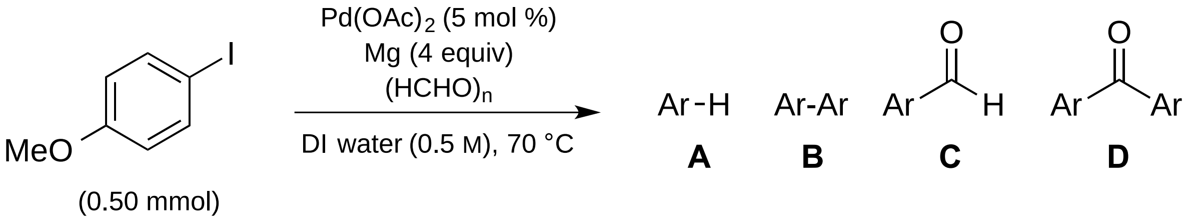

paraformaldehyde	**A**	**B**	**C**	**D**
4 equiv	07%	93%	01%	0%
8 equiv	0%	23%	35%	42%
16 equiv	0%	11%	47%	32%

Supplementary Table 1. Experiments using excess paraformaldehyde

Supplementary Methods

General Information: All reactions were carried out in a sample vial (4 mL) equipped with a Teflon-coated magnetic stir bar. Deionized water was used directly from the laboratory water system (pH 4-5). Palladium (II) acetate was purchased from Combi-blocks Inc. Magnesium powder (-50 mesh, 99+%, Lot no. 03308MQ) was purchased from Aldrich and washed prior to use, in the manner described below. Formaldehyde (37 wt % in water, stabilized with 10-15% methanol) was obtained from Acros Organics. Paraformaldehyde was purchased from Allied Chemical. All aryl halides were used without further purification. Column chromatography was carried out using silica gel 60 (230 – 400 mesh) purchased from Merck. TLC analysis was done using TLC silica gel 60 F_{254} glass plates, purchased from Merck. GCMS data were recorded on Agilent Technologies 7890A GC system coupled with Agilent Technologies 5975C mass spectrometer using HP-5MS column (30 m x 0.250 mm, 0.25 µ) purchased from Agilent Technologies. ^{1}H and ^{13}C NMR spectra were obtained in $CDCl_3$ using a 500 MHz Varian NMR spectrometer.

Experimental Section

General procedure for washing of aged magnesium

Caution! The following process is extremely vigorous and generates high volumes of nitrogen dioxide and dihydrogen. Proper precautions are to be observed.

To a beaker containing magnesium (~15 g) was slowly added concentrated nitric acid (~10 mL). A vigorous reaction ensued with the generation of nitrogen dioxide. The contents were stirred for 5-10 seconds and then were treated with deionized water (20 mL). The contents were stirred again and the water decanted. The process was repeated 4-5 times until the desired shine was obtained on the metal. The magnesium was then washed with copious amounts of deionized water several times, followed by acetone. A slurry of the metal in acetone was transferred to a round-bottomed flask and the metal dried *in vacuo* overnight. The magnesium thus obtained was used for all subsequent experiments.

This process can be avoided if a fresh bottle of magnesium is used.

General procedure for homocouplings catalyzed by $Pd(OAc)_2$

Caution! The following reaction generates varying volumes of dihydrogen depending on the grade of the Pd catalyst and magnesium. In general, all reactions involving an elemental metal, a transition metal salt and water can get extremely vigorous to explosive and can also generate obnoxiously high volumes of dihydrogen at times. Hence, all these reactions should be initially carried out on a small scale (metal powder ~ 10 mg). Also, proper precautions are to be observed.

To a sample vial (4 mL) equipped with a Teflon-coated magnetic stir bar were added in sequence $Pd(OAc)_2$ (25 μmol), magnesium powder (2.0 mmol), aryl halide (0.5 mmol), and paraformaldehyde (2.0 mmol). To the mixture was then added deionized water (1 mL). (**Caution!** The water should be added slowly, and the addition should be paused if a vigorous generation of dihydrogen ensues). The vial was covered with a phenolic cap and stirred on a reaction-block, preheated to 70 °C, for 12 h. The resulting mixture was cooled to rt, extracted with EtOAc, and then passed through a short pad of silica gel. The organic extract was concentrated *in vacuo* and purified by flash chromatography over silica gel with Et_2O/hexanes to obtain pure product.

General procedure for hetero-cross-couplings catalyzed by $Pd(OAc)_2$

To a sample vial (4 mL) equipped with a Teflon-coated magnetic stir bar were added in sequence $Pd(OAc)_2$ (25 μmol), magnesium powder (2.5 mmol), Ar-I (0.25 mmol) and Ar'-I (0.50 mmol). To the mixture was added formaldehyde (37 wt % in water, 1 mL). **Caution!** The formaldehyde should be added slowly, and the addition should be paused if a vigorous generation of dihydrogen ensues. The vial was capped and stirred on a reaction block, preheated to 70 °C, for 24 h. The resulting mixture was cooled to rt, extracted with EtOAc, and passed through a short

pad of silica-gel. The organic extract was concentrated *in vacuo* and purified by flash chromatography over silica gel with Et_2O/hexanes to obtain pure product.

Analytical Data

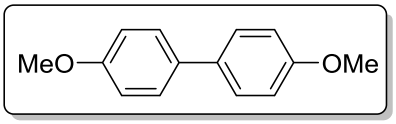

4,4'-Dimethoxy-1,1'-biphenyl (Table 2, entry 1).[1] Flash chromatography afforded the product as a white solid (**2aa**, yield 87%; **2ab**, yield 81%) .

^{1}H NMR (500 MHz, $CDCl_3$): δ 7.52 – 7.44 (m, 2H), 6.99 – 6.91 (m, 2H), 3.84 (s, 3H).

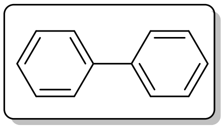

Biphenyl (Table 2, entry 2).[2] Flash chromatography afforded the product as a white solid (**2ba**, yield 80%; **2bb**, yield 75%).

^{1}H NMR (500 MHz, $CDCl_3$) δ 7.62 – 7.58 (m, 2H), 7.47 – 7.42 (m, 2H), 7.38 – 7.33 (m, 1H).

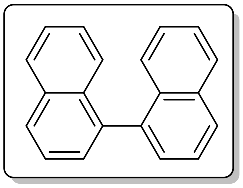

1,1'-Binaphthalene (Table 2, entry 3).[3] Flash chromatography afforded the product as a white solid (yield 83%).

^{1}H NMR (500 MHz, $CDCl_3$) δ 7.95 (dd, *J* = 8.2, 4.7 Hz, 2H), 7.60 (dd, *J* = 8.2, 7.0 Hz, 1H), 7.51 – 7.45 (m, 2H), 7.40 (d, *J* = 8.5 Hz, 1H), 7.31 – 7.27 (m, 1H).

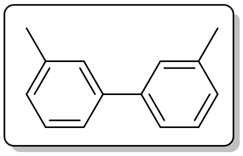

3,3'-Dimethyl-1,1'-biphenyl (Table 2, entry 4).[4] Flash chromatography afforded the product as a colorless oil (yield 79%). When the reaction was run with magnesium from a freshly purchased bottle, without further washing, the yield was 74%.

^{1}H NMR (500 MHz, $CDCl_3$) δ 7.41 – 7.37 (m, 2H), 7.32 (t, *J* = 7.5 Hz, 2H), 7.16 (d, *J* = 7.4 Hz, 1H), 2.42 (s, 3H).

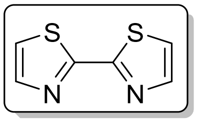

2,2'-Bithiazole (Table 2, entry 5).[5] Flash chromatography afforded the product as a white solid (yield 31%).

^{1}H NMR (500 MHz, $CDCl_3$) δ 7.90 (d, *J* = 3.2 Hz, 1H), 7.44 (d, *J* = 3.2 Hz, 1H).

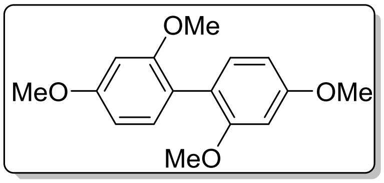

2,2',4,4'-Tetramethoxy-1,1'-biphenyl (Table 2, entry 6). [6] Flash chromatography afforded the product as a white solid (yield 53%).

^{1}H NMR (500 MHz, $CDCl_3$) δ 7.16 – 7.12 (m, 4H), 6.55 (s, 6H), 6.53 (d, J = 2.4 Hz, 2H), 3.84 (s, 13H), 3.75 (s, 13H).

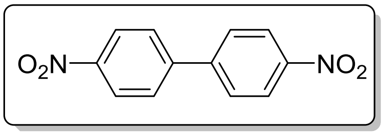

4,4'-Dinitro-1,1'-biphenyl (Table 2, entry 7).[1] Flash chromatography afforded the product as a white solid (yield 39%).

^{1}H NMR (500 MHz, $CDCl_3$) δ 8.37 – 8.34 (m, 1H), 7.81 – 7.76 (m, 1H).

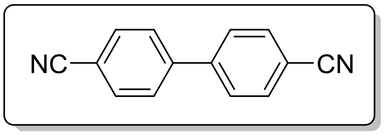

[1,1'-Biphenyl]-4,4'-dicarbonitrile (Table 2, entry 8).[1] Flash chromatography afforded the product as a white solid (yield 28%).

^{1}H NMR (500 MHz, $CDCl_3$) δ 7.80 – 7.76 (m, 1H), 7.71 – 7.67 (m, 1H).

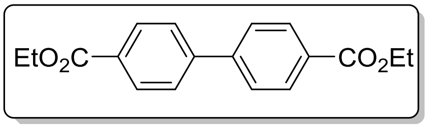

Diethyl [1,1'-biphenyl]-4,4'-dicarboxylate (Table 2, entry 9).[1] Flash chromatography afforded the product as a white solid (**bromide**, yield 52%; **iodide**, 41%).

^{1}H NMR (500 MHz, $CDCl_3$) δ 8.16 – 8.12 (m, 1H), 7.71 – 7.66 (m, 1H), 4.41 (q, J = 7.1 Hz, 2H), 1.42 (t, J = 7.1 Hz, 3H).

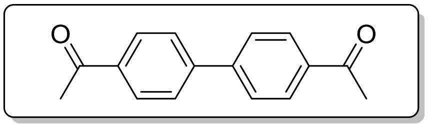

1,1'-([1,1'-Biphenyl]-4,4'-diyl)bis(ethan-1-one) (Table 2, entry 10).[1] Flash chromatography afforded the product as a white solid (**bromide**, yield 37%; **iodide**, 25%). When the reaction on

the bromide was run with magnesium from a freshly purchased bottle, without further washing, the yield was 46%.

^{1}H NMR (500 MHz, $CDCl_3$) δ 8.09 – 8.03 (m, 1H), 7.76 – 7.67 (m, 1H), 2.65 (s, 3H).

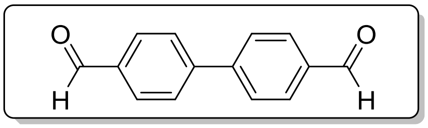

[1,1'-Biphenyl]-4,4'-dicarbaldehyde (Table 3, entry 11).[7] Flash chromatography afforded the product as a white solid (yield 71%). The reaction was run using magnesium from a freshly purchased bottle without further washing.

^{1}H NMR (400 MHz, $CDCl_3$) δ 10.08 (s, 1H), 7.99 (d, *J* = 8.3 Hz, 2H), 7.79 (d, *J* = 8.3 Hz, 2H). (Table 2, entry 11).

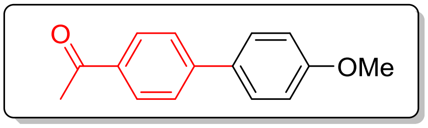

1-(4'-Methoxy-[1,1'-biphenyl]-4-yl)ethan-1-one (Table 3, entry 1).[8] Flash chromatography afforded the product as a white solid (yield 51%).

^{1}H NMR (500 MHz, $CDCl_3$) δ 8.02 – 7.99 (m, 2H), 7.66 – 7.63 (m, 2H), 7.60 – 7.55 (m, 1H), 7.02 – 6.98 (m, 1H), 3.86 (s, 3H), 2.63 (s, 3H).

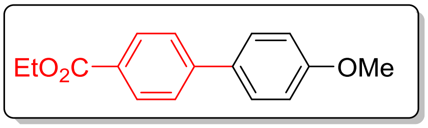

Ethyl 4'-methoxy-[1,1'-biphenyl]-4-carboxylate (Table 3, entry 2).[9] Flash chromatography afforded the product as a white solid (yield 37%).

^{1}H NMR (500 MHz, $CDCl_3$) δ 8.13 – 8.03 (m, 2H), 7.63 – 7.60 (m, 2H), 7.59 – 7.56 (m, 2H), 7.02 – 6.97 (m, 2H), 4.40 (q, *J* = 7.1 Hz, 2H), 3.86 (s, 3H), 1.41 (t, *J* = 7.1 Hz, 3H).

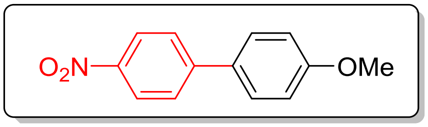

4-Methoxy-4'-nitro-1,1'-biphenyl (Table 3, entry 3).[10] Flash chromatography afforded the product as a white solid (yield 63%).

^{1}H NMR (500 MHz, $CDCl_3$) δ 8.35 – 8.19 (m, 2H), 7.71 – 7.68 (m, 2H), 7.60 – 7.56 (m, 2H), 7.04 – 7.00 (m, 2H), 3.88 (s, 3H).

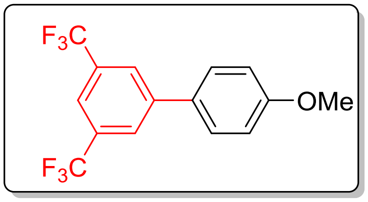

4'-Methoxy-3,5-bis(trifluoromethyl)-1,1'-biphenyl (Table 3, entry 4).[11] Flash chromatography afforded the product as a white solid (yield 48%).

^{1}H NMR (500 MHz, $CDCl_3$) δ 7.97 (s, 2H), 7.80 (s, 1H), 7.58 – 7.52 (m, 2H), 7.05 – 7.00 (m, 2H), 3.88 (s, 3H).

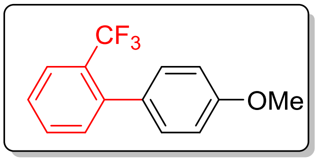

4'-Methoxy-2-(trifluoromethyl)-1,1'-biphenyl (Table 3, entry 5).[12] Flash chromatography afforded the product as yellowish brown oil (yield 33%).

^{1}H NMR (500 MHz, $CDCl_3$) δ 7.74 (s, 1H), 7.54 (t, J = 7.5 Hz, 1H), 7.44 (t, J = 7.6 Hz, 1H), 7.33 (d, J = 7.2 Hz, 1H), 7.28 – 7.24 (m, 2H), 6.96 – 6.91 (m, 2H), 3.86 (s, 3H).

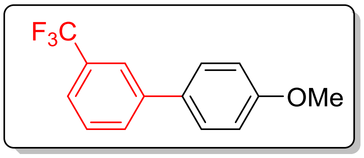

4'-Methoxy-3-(trifluoromethyl)-1,1'-biphenyl (Table 3, entry 6).[11] Flash chromatography afforded the product as a colorless oil (yield 21%).

^{1}H NMR (500 MHz, $CDCl_3$) δ 7.79 (s, 1H), 7.72 (d, J = 7.3 Hz, 1H), 7.57 – 7.50 (m, 4H), 7.02 – 6.98 (m, 2H), 3.87 (s, 3H).

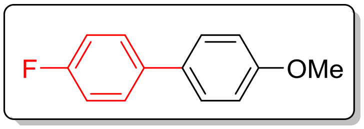

4-Fluoro-4'-methoxy-1,1'-biphenyl (Table 3, entry 7).[13] Flash chromatography afforded the product as a white solid (yield 41%).

^{1}H NMR (500 MHz, $CDCl_3$) δ 7.52 – 7.46 (m, 4H), 7.13 – 7.08 (m, 2H), 7.00 – 6.96 (m, 2H), 3.85 (s, 3H).

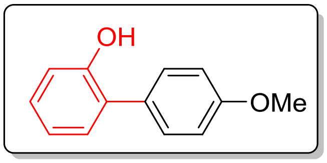

4'-Methoxy-[1,1'-biphenyl]-2-ol (Table 3, entry 8).[14] Flash chromatography afforded the product as a yellowish-white solid (yield 61%).

^{1}H NMR (500 MHz, $CDCl_3$) δ 7.42 – 7.37 (m, 2H), 7.23 (ddd, J = 9.5, 7.5, 3.9 Hz, 2H), 7.05 – 7.01 (m, 2H), 6.98 (tt, J = 3.8, 1.1 Hz, 2H), 5.18 (s, 1H), 3.86 (s, 3H).

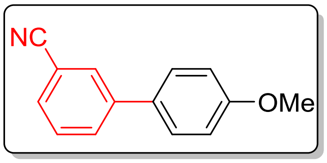

4'-Methoxy-[1,1'-biphenyl]-3-carbonitrile (Table 3, entry 9).[15] Flash chromatography afforded the product as a white solid (yield 54%). When the reaction for the bromide was run with magnesium from a freshly purchased bottle, without further washing, the yield was 50%.

^{1}H NMR (500 MHz, $CDCl_3$) δ 7.83 – 7.81 (m, 1H), 7.77 (ddd, J = 7.8, 1.9, 1.3 Hz, 1H), 7.57 (dt, J = 7.7, 1.4 Hz, 1H), 7.51 – 7.48 (m, 3H), 7.02 – 6.99 (m, 2H), 3.86 (s, 3H).

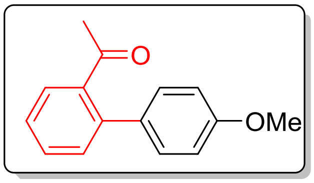

1-(4'-Methoxy-[1,1'-biphenyl]-2-yl)ethan-1-one (Table 3, entry 10).[16] Flash chromatography afforded the product as a white solid (yield 17%).

^{1}H NMR (500 MHz, $CDCl_3$) δ 7.54 – 7.46 (m, 2H), 7.38 (dd, J = 11.8, 4.4 Hz, 2H), 7.29 – 7.24 (m, 2H), 6.99 – 6.94 (m, 2H), 3.86 (s, 3H), 2.01 (s, 3H).

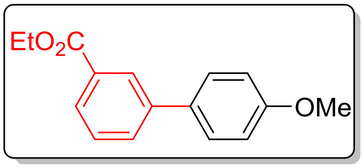

Ethyl 4'-methoxy-[1,1'-biphenyl]-3-carboxylate (Table 3, entry 11).[9] Flash chromatography afforded the product as a colorless oil (yield 47%).

^{1}H NMR (500 MHz, $CDCl_3$) δ 8.24 (t, J = 1.8 Hz, 1H), 8.00 – 7.95 (m, 1H), 7.74 (ddd, J = 7.7, 1.9, 1.2 Hz, 1H), 7.59 – 7.54 (m, 2H), 7.51 – 7.45 (m, 1H), 7.02 – 6.97 (m, 2H), 4.41 (q, J= 7.1 Hz, 2H), 3.86 (s, 3H), 1.42 (t, J= 7.1 Hz, 3H).

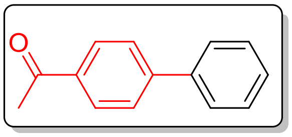

1-([1,1'-Biphenyl]-4-yl)ethan-1-one (Table 3, entry 12).[17] Flash chromatography afforded the product as a white solid (yield 58%).

^{1}H NMR (500 MHz, $CDCl_3$) δ 8.04 (d, J = 8.2 Hz, 2H), 7.69 (d, J = 8.2 Hz, 2H), 7.63 (d, J = 7.6 Hz, 2H), 7.48 (t, J = 7.7 Hz, 2H), 7.44 – 7.38 (m, 1H), 2.64 (s, 3H).

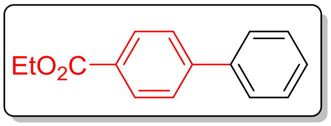

Ethyl [1,1'-biphenyl]-4-carboxylate (Table 3, entry 13).[18] Flash chromatography afforded the product as a white solid (yield 43%).

^{1}H NMR (500 MHz, $CDCl_3$) δ 8.12 (dt, *J* = 8.5, 1.8 Hz, 2H), 7.72 – 7.60 (m, 4H), 7.53 – 7.43 (m, 2H), 7.44 – 7.36 (m, 1H), 4.41 (q, *J* = 7.1 Hz, 2H), 1.42 (t, *J* = 7.1 Hz, 3H).

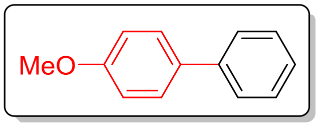

4-Methoxy-1,1'-biphenyl (Table 3, entry 14).[14] Flash chromatography afforded the product as a white solid (yield 45%).

^{1}H NMR (500 MHz, $CDCl_3$) δ 7.58 – 7.50 (m, 4H), 7.45 – 7.38 (m, 2H), 7.34 – 7.26 (m, 1H), 7.02 – 6.95 (m, 2H), 3.85 (s, 3H).

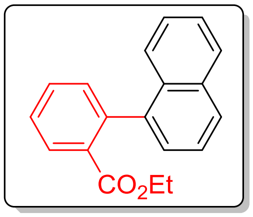

Ethyl 2-(naphthalen-1-yl)benzoate (Table 3, entry 15).[19] Flash chromatography afforded the product as a colorless oil (yield 31%).

^{1}H NMR (500 MHz, $CDCl_3$) δ 8.07 – 8.03 (m, 1H), 7.87 (dd, *J* = 12.0, 8.0 Hz, 2H), 7.61 (t, *J* = 7.5 Hz, 1H), 7.56 – 7.30 (m, 7H), 3.84 – 3.71 (m, 2H), 0.56 – 0.51 (t, J= 7.1 Hz, 3H).

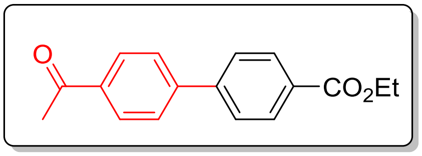

Ethyl 4'-acetyl-[1,1'-biphenyl]-4-carboxylate (Table 3, entry 16).[20] Flash chromatography afforded the product as a white solid (yield 17%).

^{1}H NMR (500 MHz, $CDCl_3$) δ 8.16 – 8.12 (m, 2H), 8.07 – 8.04 (m, 2H), 7.74 – 7.67 (m, 4H), 4.42 (q, *J* = 7.1 Hz, 2H), 2.6 (s, 3H), 1.42 (t, *J* = 7.1 Hz, 3H).

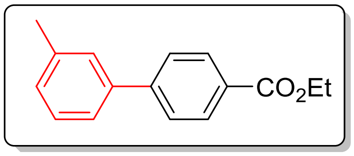

Ethyl 3'-methyl-[1,1'-biphenyl]-4-carboxylate (Table 3, entry 17).[21] Flash chromatography afforded the product as a colorless oil (yield 35%).

^{1}H NMR (500 MHz, $CDCl_3$) δ 8.12 – 8.08 (m, 2H), 7.67 – 7.62 (m, 2H), 7.43 (d, *J* = 9.5 Hz, 2H), 7.36 (d, *J* = 7.5 Hz, 1H), 7.21 (d, *J* = 7.5 Hz, 1H), 4.40 (q, *J* = 7.1 Hz, 2H), 2.43 (s, 3H), 1.42 (t, *J* = 7.1 Hz, 3H).

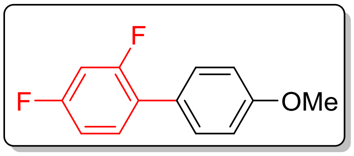

2,4-Difluoro-4'-methoxy-1,1'-biphenyl (Table 3, entry 18).[22] Flash chromatography afforded the product as a white solid (yield 52%).

^{1}H NMR (500 MHz, $CDCl_3$) δ 7.46 – 7.41 (m, 2H), 7.40 – 7.33 (m, 1H), 7.00 – 6.96 (m, 2H), 6.95 – 6.86 (m, 2H), 3.86 (s, 3H).

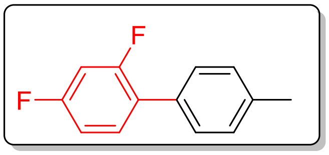

2,4-Difluoro-4'-methyl-1,1'-biphenyl (Table 3, entry 19).[23] Flash chromatography afforded the product as a colorless oil (yield 46%).

^{1}H NMR (500 MHz, $CDCl_3$) δ 7.42 – 7.36 (m, 3H), 7.26 (dd, *J* = 1.5, 0.9 Hz, 2H), 6.96 – 6.87 (m, 2H), 2.40 (s, 3H).

Supplementary References

1 Nising, C. F., Schmid, U. K., Nieger, M. & Bräse, S. A New Protocol for the One-Pot Synthesis of Symmetrical Biaryls. *J. Org. Chem.* **69**, 6830-6833 (2004).

2 Bandari, R., Höche, T., Prager, A., Dirnberger, K. & Buchmeiser, M. R. Ring-Opening Metathesis Polymerization Based Pore-Size-Selective Functionalization of Glycidyl Methacrylate Based Monolithic Media: Access to Size-Stable Nanoparticles for Ligand-Free Metal Catalysis. *Chem. Eur. J.* **16**, 4650-4658 (2010).

3 Viswanathan, G. S., Wang, M. & Li, C.-J. A Highly Regioselective Synthesis of Polysubstituted Naphthalene Derivatives through Gallium Trichloride Catalyzed Alkyne–Aldehyde Coupling. *Angew. Chem. Int. Ed.* **41**, 2138-2141 (2002).

4 Chao, C. S., Cheng, C. H. & Chang, C. T. New method for the preparation of activated nickel and cobalt powders and their application in biaryl synthesis. *J. Org. Chem.* **48**, 4904-4907 (1983).

5 Truong, T., Alvarado, J., Tran, L. D. & Daugulis, O. Nickel, Manganese, Cobalt, and Iron-Catalyzed Deprotonative Arene Dimerization. *Org. Lett.* **12**, 1200-1203 (2010).

6 Goto, H., Furusho, Y., Miwa, K. & Yashima, E. Double Helix Formation of Oligoresorcinols in Water: Thermodynamic and Kinetic Aspects. *J. Am. Chem. Soc.* **131**, 4710-4719 (2009).

7 Kirai, N. & Yamamoto, Y. Homocoupling of Arylboronic Acids Catalyzed by 1,10-Phenanthroline-Ligated Copper Complexes in Air. *Eur. J. Org. Chem.* **2009**, 1864-1867 (2009).

8 Bernhardt, S., Manolikakes, G., Kunz, T. & Knochel, P. Preparation of Solid Salt-Stabilized Functionalized Organozinc Compounds and their Application to Cross-Coupling and Carbonyl Addition Reactions. *Angew. Chem. Int. Ed.* **50**, 9205-9209 (2011).

9 Gavryushin, A., Kofink, C., Manolikakes, G. & Knochel, P. Efficient Cross-Coupling of Functionalized Arylzinc Halides Catalyzed by a Nickel Chloride−Diethyl Phosphite System. *Org. Lett.* **7**, 4871-4874 (2005).

10 Felpin, F.-X. & Fouquet, E. Efficient and Practical Cross-Coupling of Arenediazonium Tetrafluoroborate Salts with Boronic Acids Catalyzed by Palladium(0)/Barium Carbonate. *Adv. Synth. Catal.* **350**, 863-868 (2008).

11 Ackermann, L. & Althammer, A. Air-Stable PinP(O)H as Preligand for Palladium-Catalyzed Kumada Couplings of Unactivated Tosylates. *Org. Lett.* **8**, 3457-3460 (2006).

12 Bonin, H., Delbrayelle, D., Demonchaux, P. & Gras, E. Base free aryl coupling of diazonium compounds and boronic esters: self-activation allowing an overall highly practical process. *Chem. Commun.* **46**, 2677-2679 (2010).

13 Döbele, M., Vanderheiden, S., Jung, N. & Bräse, S. Synthesis of Aryl Fluorides on a Solid Support and in Solution by Utilizing a Fluorinated Solvent. *Angew. Chem. Int. Ed.* **49**, 5986-5988 (2010).

14 Ackermann, L., Kapdi, A. R., Fenner, S., Kornhaaß, C. & Schulzke, C. Well-Defined Air-Stable Palladium HASPO Complexes for Efficient Kumada–Corriu Cross-Couplings of (Hetero)Aryl or Alkenyl Tosylates. *Chem. Eur. J.* **17**, 2965-2971 (2011).

15 Papoian, V. & Minehan, T. Palladium-Catalyzed Reactions of Arylindium Reagents Prepared Directly from Aryl Iodides and Indium Metal. *J. Org. Chem.* **73**, 7376-7379 (2008).
16 Lipshutz, B. H., Butler, T. & Swift, E. C−C Bond Formation Catalyzed Heterogeneously by Nickel-on-Graphite (Ni/Cg). *Org. Lett.* **10**, 697-700 (2008).
17 Alacid, E. & Nájera, C. First Cross-Coupling Reaction of Potassium Aryltrifluoroborates with Organic Chlorides in Aqueous Media Catalyzed by an Oxime-Derived Palladacycle†. *Org. Lett.* **10**, 5011-5014 (2008).
18 Amatore, M. & Gosmini, C. Efficient Cobalt-Catalyzed Formation of Unsymmetrical Biaryl Compounds and Its Application in the Synthesis of a Sartan Intermediate. *Angew. Chem. Int. Ed.* **47**, 2089-2092 (2008).
19 Wang, C., Rakshit, S. & Glorius, F. Palladium-Catalyzed Intermolecular Decarboxylative Coupling of 2-Phenylbenzoic Acids with Alkynes via C−H and C−C Bond Activation. *J. Am. Chem. Soc.* **132**, 14006-14008 (2010).
20 Chen, Y.-H. & Knochel, P. Preparation of Aryl and Heteroaryl Indium(III) Reagents by the Direct Insertion of Indium in the Presence of LiCl. *Angew. Chem. Int. Ed.* **47**, 7648-7651 (2008).
21 Liu, J. *et al.* Effective Pd-Nanoparticle (PdNP)-Catalyzed Negishi Coupling Involving Alkylzinc Reagents at Room Temperature. *Org. Lett.* **10**, 2661-2664 (2008).
22 Barder, T. E., Walker, S. D., Martinelli, J. R. & Buchwald, S. L. Catalysts for Suzuki−Miyaura Coupling Processes: Scope and Studies of the Effect of Ligand Structure. *J. Am. Chem. Soc.* **127**, 4685-4696 (2005).
23 Asachenko, A. F., Sorochkina, K. R., Dzhevakov, P. B., Topchiy, M. A. & Nechaev, M. S. Suzuki–Miyaura Cross-Coupling under Solvent-Free Conditions. *Adv. Synth. Catal.* **355**, 3553-3557 (2013).
